# Unveiling Activation Process of C═N Cathode for High‐Performing Zinc‐Organic Batteries

**DOI:** 10.1002/advs.76326

**Published:** 2026-06-28

**Authors:** Xiaodong Geng, Zhangyu Wang, Weiyao Bi, Qian Zhao, Hongting Ma, Kai Yang, Ying Liu, Zhiqian Li, Qinghua Deng, Dan Zhao, Yuqian Jiang, Nan Zhu

**Affiliations:** ^1^ School of Chemistry Dalian University of Technology Dalian Liaoning China; ^2^ Laboratory of Organic Electronics Department of Science and Technology Linköping University Norrköping Sweden; ^3^ Key Laboratory of Nanosystem and Hierarchical Fabrication CAS Center for Excellence in Nanoscience National Center for Nanoscience and Technology Beijing China

**Keywords:** activation phenomenon, DPPT, long‐cycling life, organic cathode, zinc‐ion batteries

## Abstract

Zinc‐organic batteries (ZOBs) based on C═N compounds arouse great interest due to wide structure, high capacity, and long cycle life. However, activation phenomenon of C═N compound is seldom discussed in ZOBs, as well as activation mechanism during charging/discharging. Herein, DPPT (Diphenazine‐pyrenetetraimine) with eight C═N active sites is first synthesized for ZOBs, delivering ultralong cycling life (45 000 cycles) and good rate performance (20 C). Notably, there shows obvious activation process at beginning cycles because of the transformation of DPPT‐eight proton into DPPT. In situ experiments and DFT calculations reveal co‐participation mechanism of Zn^2+^ & H^+^ with optimized structure of 2DPPT‐1Zn‐14H deriving from 16 discharged products. Impressively, wearable NH_3_ sensors could be powered by as‐fabricated flexible DPPT ZOBs, making it possible from the restriction of traditional power supply. Prospectively, activation mechanism of high‐performing DPPT would provide methods for further design of organic structures into ZOBs and offer self‐powered energy for wearable sensors.

## Graphical abstract

A high‐performance organic cathode of DPPT has been designed for aqueous zinc‐ion batteries (ZIBs), showing ultralong cycle life of 45 000 cycles. Besides, flexible DPPT ZIBs with stable capacity are applied in wearable NH_3_ sensors. Notably, the activation mechanism of the transformation of DPPT‐eight proton into DPPT is proposed and proved which provides more insights into the mechanism of organic ZIs.

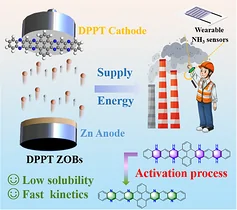

## 1 Introduction

The demand for energy storage devices, exploring high‐performing batteries, is rapidly increasing for sustainable environment [1, 2]. Among them, aqueous zinc‐ion batteries (AZIBs) play pivotal role due to their inherent safety, good electrochemical performance and environmental friendliness [3, 4]. AZIBs possess high theoretical capacity (820 mAh g^−1^) and low reduction potential (−0.76 V vs. Standard Hydrogen Electrode (SHE)) [5, 6, 7]. However, universal implementation of AZIBs is still full of limitation because of the lack of suitable cathode materials. Presently, inorganic cathode materials of AZIBs mainly include vanadium and manganese‐based oxides/sulfides, as well as Prussian blue analogs (PBAs). Yet, there faces significant challenges for constraining their practical applications. V‐based oxides/sulfides are restricted by low operating voltage, while Mn‐based oxides/sulfides are limited by poor stability [8, 9, 10]. Similarly, the inherent low capacity of PBAs hinders their development [11, 12]. In comparison, organic cathode materials have sparked researchers’ interest in developing high‐performance AZIBs, owning to wide variety, high theoretical capacity and long cycling life [13, 14, 15].

Based on advantages of organic cathode materials of AZIBs, Zn‐organic batteries (ZOBs) with high performance and good flexibility have been researched widely [16, 17, 18, 19, 20]. Up to now, cathode materials of ZOBs mainly contain C═O compounds, C═O/C═N compounds and C═N compounds. Moreover, activation phenomenon universally occurs in ZOBs. C═O compounds of ZOBs, such as Zn‐PDBS (Poly(2,5‐Dihydroxy‐1,4‐Benzoquinonyl Sulfide) battery by Zhang's group, showed obvious activation process [21]. The capacity was increased rapidly from first cycle to 200^th^ cycle and the activation reason was caused by the conversion of phenol structure into quinone structure. The phenol structure is formed by the reaction of quinone with protons during discharging and it would be gradually transformed into quinone structure, leading to more active sites and increasing capacity. Furthermore, there is also activation progress in ZOBs based on C═O/C═N compounds. For instance, Zn‐DPT(dibenzo[b,i]‐phenazine‐5,7,12,14‐tetrone) battery gradually increased and reached maximum capacity after approximately 600 cycles [22]. Resulting from the activation progress of trace dissolution−redeposition process of DPT, more porous structures and surface modification of organic electrodes make it easy for zinc and proton insertion thus capacity is increased. Besides, ZOBs based on C═N compounds have been developed since 2020. Niu and coworkers reported Zn‐HATN (hexaazatrinaphthalene) battery with long cycling life of 5000 cycles [23] and several functional groups were then introduced to HATN structure such as ─COOH, ─CN, etc [24, 25, 26]. However, the activation phenomenon has rarely been observed in ZOBs based on C═N compounds and the activation mechanism is still unknown.

Herein, an original C═N compound of DPPT (Diphenazine‐pyrenetetraimine) has been designed by one‐step reaction of PTO (Pyrenetetraone) and DAP (Diaminophenazine). There are twelve aromatic rings and eight C═N active sites in DPPT molecule, enlarging theoretical specific capacity, being stronger conjugation and π‐π stacking. As expected, fabricated DPPT ZOBs show ultralong cycling life (45 000 cycles) and good rate performance (20 C). Remarkably, there is obvious activation process and capacity is increased from 110 to 185 mAh g^−1^ at 5A g^−1^ after 400 cycles. The transformation of DPPT‐8H (formed by the reaction of DPPT with eight protons in discharged progress) into DPPT, proved by Ex situ ^1^H‐NMR and in situ Raman, is the main reason for the activation process. In addition, in situ experiments reveal the active sites of C═N and the co‐participation mechanism of Zn^2+^ & H^+^. Furthermore, DFT calculations indicate optimized structure of 2DPPT‐1Zn‐14H from two DPPT molecules binding with one zinc ion and fourteen protons after calculating 16 discharged products. Additionally, flexible DPPT ZOBs with a sandwich structure are assembled and can work at flatting, bending, beating, drilling and cutting states, indicating anti‐strike ability and anti‐drilling stability and cuttability. Besides, flexible DPPT ZOBs can supply energy for wearable NH_3_ sensor, breaking the limitation of traditional power supply. Prospectively, DPPT ZOBs with ultralong lifespan and activation mechanism of transformation from DPPT‐8H into DPPT would provide ideas into storage mechanism of ZOBs and promote their development.

## 2 Results and Discussion

### 2.1 Design and Characterizations of DPPT

Lighten by previously published C═N compounds for ZOBs cathodes, a novel C═N compound DPPT(Diphenazine‐pyrenetetraimine) had been designed with 8 active cites and strongly conjugated carbon skeleton. As shown in Figure 1a, the synthetic route was illustrated briefly as the reaction of PTO (Pyrenetetraone) with DAP (Diaminophenazine) and then purification by HNO_3_ (30 wt.%). The obtained DPPT powder with reddish‐brown color was verified by MS and ^1^H‐NMR spectra (Figure S1 and S2). The molecular ion peak at 610.16489 m/z in MS spectrum confirmed the successful synthesis of DPPT and five peaks at 8.18, 8.53, 8.79, 9.28, and 9.49 (ppm) in ^1^H‐NMR spectrum indicated five types of H atoms in the clear structure of DPPT. In FT‐TR spectrum, the peaks of 1525, 1485&1465, and 1330 cm^−1^ contributed to C═N, Ar(C═C), and C─N vibration respectively (Figure S3). Moreover, DPPT showed different peaks in XRD patterns compared to raw materials of PTO and DAP (Figure 1b), verifying the formation of a new crystal of DPPT. Notably, there was a strong peak at 26.7°, related to π–π stacking interaction of DPPT [27]. In addition, green, purple, and orange peaks in the XPS C 1s spectrum in DPPT (Figure S4) demonstrated C‐SP_2_/C‐Hx bond, C═N bond and π‐π stacking, respectively. Furthermore, the existence of C═N of DPPT was confirmed by the peaks of C═N & C─N in N1s spectra (Figure S5). As revealed by SEM images (Figure S6), the morphology of PTO was micronod and DAP was flowe‐like structure and DPPT was nanoflake. In the TEM image of DPPT (Figure 1c), the DPPT nanoflake width was around 200–300 nm with length about 500–600 nm. Besides, C and N elements were uniformly distributed in DPPT structure in the STEM mapping image.

**FIGURE 1:**
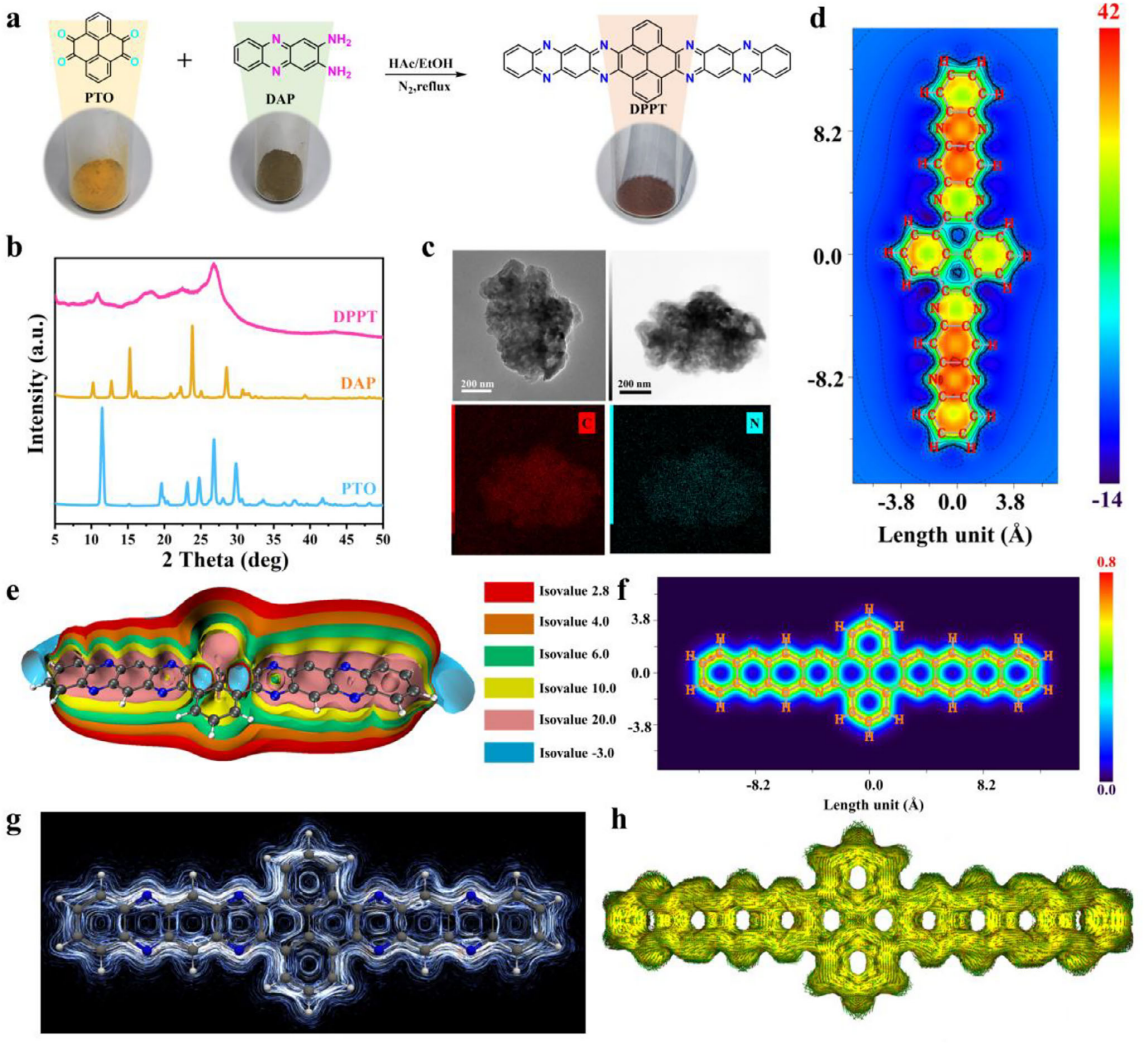
Properties of DPPT. (a) Synthetic procedure of DPPT. (b) XRD patterns of PTO, DAP and DPPT. (c) TEM image and STEM mapping of DPPT. (d) Color‐filled map of ICSS_ZZ_ of DPPT. (e) Cutting ICSS_ZZ_ of DPPT at different iso‐values. (f) LOL‐π map of DPPT. (g) GIMIC full‐space dynamic streamline field diagram of DPPT. (h) ACID plots of DPPT.

To measure the aromaticity of DPPT, ICSSzz (iso‐chemical shielding surfaces), LOL(localized orbital locator)‐π electron distribution, GIMIC (gauge including magnetically induced current method) and ACID (anisotropy of the induced current density) were performed [28, 29]. DPPT showed a high value in red & yellow colors in ICSS_ZZ_ color‐filled map, indicating good aromaticity with high stability (Figure 1d). In addition, green iso‐surface with positive Z‐component shielding values enveloped the DPPT molecule and most rings still were filled with a high pink iso‐surface of 20, meaning strong aromaticity (Figure 1e and Figure S7). Besides, DPPT possessed a highly π‐conjugated system with efficient electron delocalization in LOL‐π color‐filled map and grid map (Figure 1f and Figure S8). Compared to PTO and DAP (Figures S9 and S10), more π‐electrons were delocalized in DPPT, resulting in more stability of DPPT. Moreover, continuous current flows were through the entire backbone of DPPT using the GIMIC method, proving global π‐aromaticity (Figure 1g, Figure S11, Video S1). Furthermore, diamagnetic current flows in ACID plots (Figure 1h) also demonstrated the high aromaticity of DPPT. In summary, it is the good aromaticity of DPPT that makes it very stable during charging and discharging with a long cycling life of 45 000 (Figure 2e).

**FIGURE 2:**
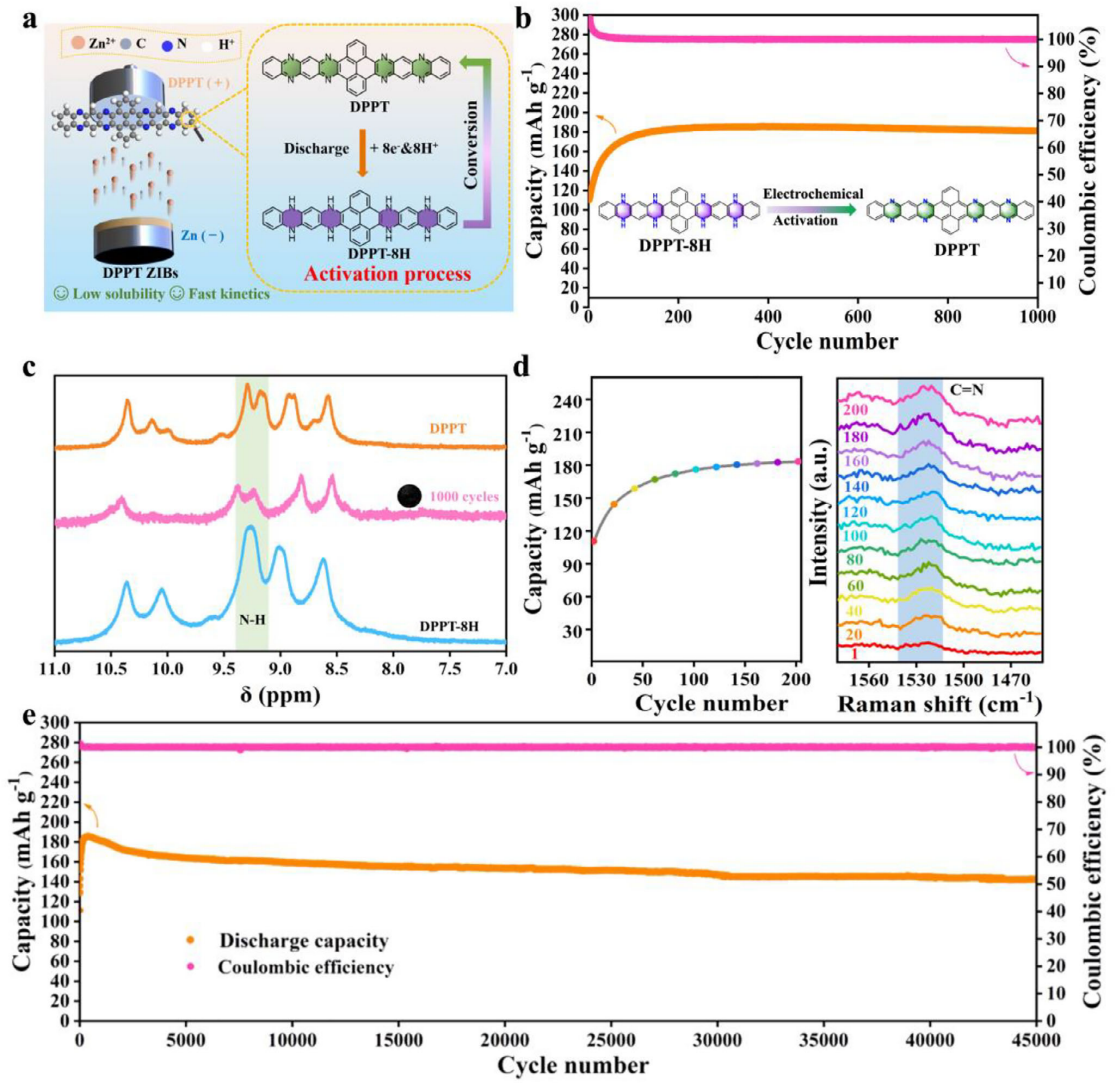
Activation mechanism of DPPT ZOBs during cycles. (a) Schematic diagram of DPPT ZOBs during activation process. (b) Cycling performance and activation mechanism of DPPT ZOBs. (c) Ex situ ^1^H‐NMR of DPPT‐8H and DPPT cathodes. (d) In situ Raman spectra of DPPT ZOBs at fully charged state during the first 200 cycles. (e) Long‐term cycling performance of DPPT ZOBs at 5 A g^−1^.

### 2.2 Activation Mechanism and Kinetic & Electrochemical Performance of DPPT ZOBs

For measuring electrochemical performances, DPPT ZOBs were fabricated by DPPT cathode, zinc anode, and 3.5 M Zn(ClO_4_)_2_ electrolyte (Figure 2a). Notably, there was an obvious activation process of cycling performance of DPPT ZOBs at 5 A g^−1^ (Figure 2b). The initial capacity was 110 mAh g^−1^ then increased into 185 mAh g^−1^ after 400 cycles. The main reason of activation process was the transformation of DPPT‐8H (formed by reaction of DPPT with eight protons during initial discharged process) into DPPT. The discharged capacity was high of 136 mAh g^−1^ in initial cycle (Figure S12), yet the charged capacity at first cycle was low of 96 mAh g^−1^, meaning not all DPPT‐8H converted to DPPT at first charging progress and the remaining DPPT‐8H would gradually convert to DPPT during subsequent charging process. To prove the conversion of DPPT‐8H into DPPT, pure DPPT‐8H was synthesized by reduction of DPPT by NaBH_4_ reagent (Figure S13). The obtained DPPT‐8H was black powder and the peak at 3380 cm^−1^ of FT‐IR spectrum indicated the exitance of N‐H in DPPT‐8H (Figure S14). Besides, the clear structure and successful synthesis of DPPT‐8H were proved by the molecular ion peak at 618.22700 m/z in MS spectrum (Figure S15).

Then DPPT‐8H ZOBs were assembled to obtain DPPT‐8H cathodes in different cycles of 200, 500 and 1000. Initial DPPT & DPPT‐8H cathodes and DPPT‐8H cathodes in different cycles were dissolved in DMSO‐D_6_ reagent to test ex situ ^1^H‐NMR (Figure 2c and Figure S16). In ^1^H‐NMR spectra, the DPPT‐8H at the initial state showed strong peak at 9.3 ppm of N─H bond then it disappeared after 1000 cycles. Besides, the peak shape of DPPT‐8H was more similar to DPPT from 200 to 1000 cycles, indicating the conversion of DPPT‐8H to DPPT during electrochemical cycling. Moreover, the intensity of C═N at 1525 cm^−1^ was increased gradually at fully charged status during the first 200 cycles from in situ Raman spectra (Figure 2d), also verifying the transformation of DPPT‐8H to DPPT (the key reason for activation process), leading to more active sites and increasing capacity. Additionally, there was no activation at low current densities of 0.1, 0.2, and 0.5 A g^−1^, yet activation appeared at high current densities of 1, 2, and 5A g^−1^ (Figures S17 and S18). To figure out the reason of this phenomenon, the energy required to the de‐intercalation of per H^+^ or Zn^2+^ was calculated. As shown in Figure S19, the energy required to de‐intercalation of H^+^ (365.56111 kcal mol^−1^) was much higher than Zn^2+^ (331.63532 kcal mol^−1^) during charging process, revealing that de‐intercalation of H^+^ was need of more energy and the DPPT‐8H was not easy to transfer to DPPT at low current densities. Thus, there was no activation at low current densities and no activation process was observed under low current density conditions in rate performance (Figure S28). Besides, there was no activation process in DPPT ZOBs, using saturated Zn(ClO_4_)_2_/Acetonitrile electrolyte (not containing any H^+^) at high current densities of 5A g^−1^, proving that the activation process was not caused by the structural transformation of Zinc‐containing discharging products (Figure S20). Hence, the gradually transformation of DPPT‐8H into DPPT was the key reason of activation at high current densities.

Furthermore, dynamic analysis was performed. The current response is composed of diffusion progress and capacitive progress, as depicted in Equation (1) [30, 31],

(1) $$i=k_{1}v+k_{2}v^{\frac{1}{2}}$$

where *k_1_* &*k_2_* are constants, *i* is current at a fixed potential (V), *k_2_υ* ^1/2^ means the current of diffusion progress and *k_1_υ* means the current of capacitive progress. There was 86% capacitive contribution at 1 mV s^−1^, resulting in good rate performance (Figure S21). In addition, the capacitance contribution of DPPT ZOBs increased from 72% to 86% when scan rate increased from 0.2 to 1 mV s^−1^ (Figure S22), indicating the leading role of capacitance progress at high scan rates. Besides, there was one pair of main peaks in CV curves at different scan rates (Figure S23). The main peaks at 0.91/0.80 V are attributed to the reaction of C═N with zinc ions and protons. Moreover, the relationship between the current (*i*) and scan rate (*ν*) is illustrated by Equation (2) [32, 33],

(2) $$i=av^{b}$$

where a and b are constants and capacity is controlled by the capacitance when b is near to 1 and capacity is dominated by a diffusion process while b is closed to 0.5 [34, 35]. The b values of main peaks were 0.89 and 0.87, respectively (Figure S23). The b values were all near to 1, indicating capacity was mainly controlled by the capacitance and fast kinetics. Furthermore, the galvanostatic intermittent titration technique (GITT) was studied (Figures S24‐S27). During charging/discharging process, the diffusion coefficient (D) of Zn^2+^ first decreased, meaning fast diffusion process in initial [36, 37]. Besides, there were minimum values near the charging/discharging platform of 0.65–0.95 V. In addition, DPPT ZOBs showed a high capacity of 286 mAh g^−1^ at 0.1C (351 mA g^−1^), and there was still a capacity of 271 mAh g^−1^ when it came back from 20C to 0.1C, verifying good rate performance because of fast dynamics (Figure S28). Due to the activation process, enhanced π‐π stacking and good aromaticity of DPPT molecules, fabricated DPPT ZOBs demonstrated super‐long cycling life of 45 000 cycles with 83% capacity retention at 5A g^−1^ (Figure 2e), which will promote the development of organic zinc‐ion batteries toward long lifespan and high capacity.

### 2.3 Theoretical Calculations

To evaluate the specific active sites and verify the optimal structure during discharged process, density functional theory (DFT) calculations were computed. The C═O of PTO and C═N of DAP & DPPT showed low ESP values in red/orange region, indicating more electrons distributed in C═O & C═N (Figure 3a). Because of more electron distribution, C═O of PTO and C═N of DAP & DPPT were easier to react with Zn^2+^ and H^+^, verifying the active sites of C═O in PTO and C═N in DAP & DPPT. Additionally, DPPT possessed a lower energy gap (2.5510 eV) than PTO (3.5189 eV) and DAP (3.5192 eV), verifying fast electron transmission and better electrical conductivity (Figure 3b). Thus, DPPT ZOBs showed a good rate performance. To calculate the optical energy gap (E_g_), the UV–vis spectra were performed (Figures S29–S31). DPPT possessed a low E_g_ value of 2.61 eV (Figure 3c) due to the π‐conjugated superstructure of DPPT and enhanced π‐π interaction of DPPT molecules. The lower E_g_ values (2.61 eV) of DPPT than DAP (3.16 eV) and PTO (3.99 eV) indicated higher inherent electronical conductivity, which promoted redox reaction with low energy barriers [38].

**FIGURE 3:**
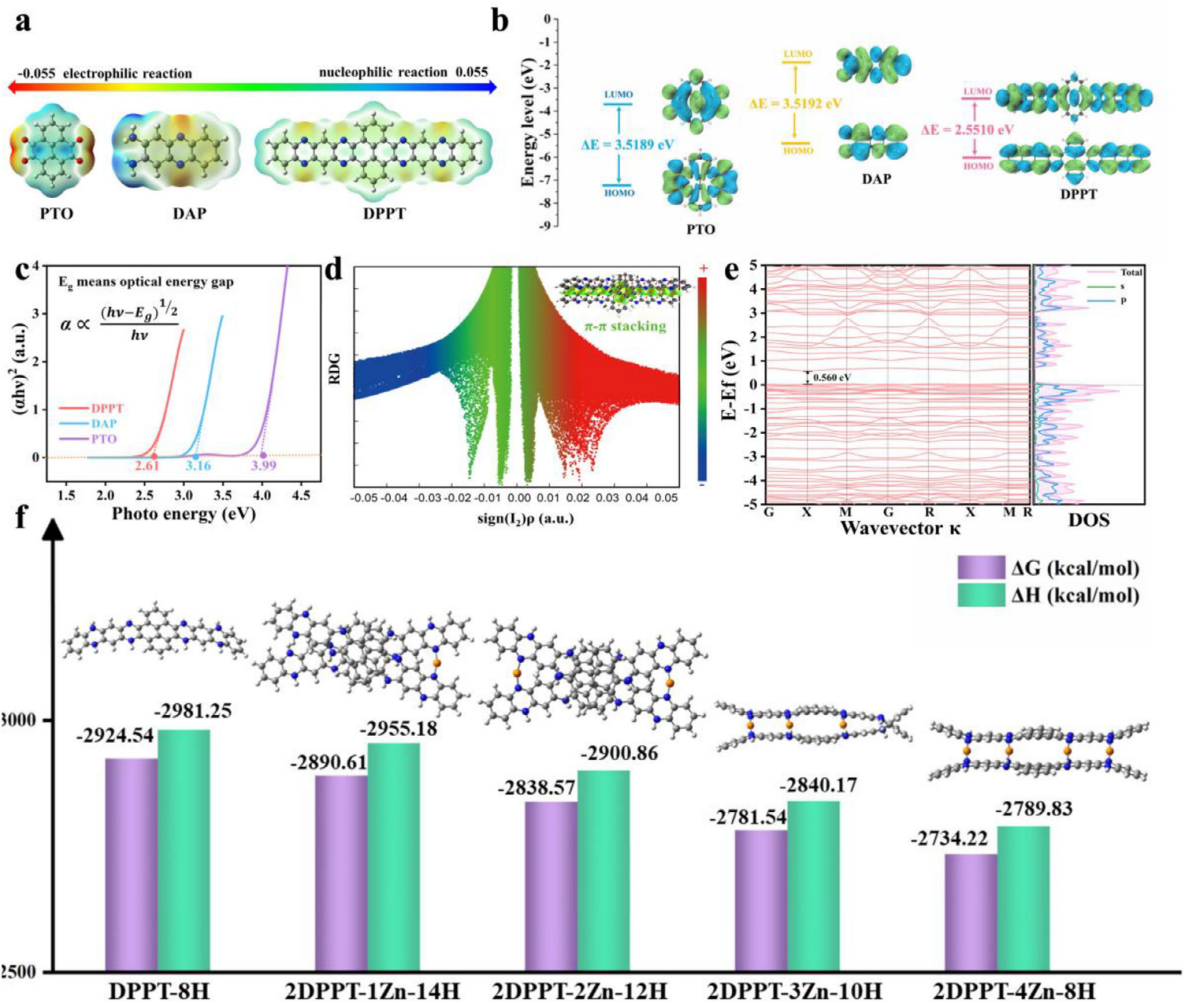
Theoretical calculations of DPPT ZOBs. (a) The ESP (Electronic Static Potential) mapped molecular surface of PTO, DAP and DPPT. (b) Calculated HOMO/LUMO energy levels and orbit distributions of PTO, DAP and DPPT. (c) Calculated optical energy gaps (E_g_) of PTO, DAP and DPPT. (d) RDG (Reduced density gradient) scatter maps *vs*. sign(λ_2_)ρ and visualization of DPPT. (e) Band structure spectrum and corresponding partial density of state (PDOS) of DPPT. (f) The histogram of binding Gibbs free energy (ΔG) & binding enthalpy (ΔH) per DPPT of optimal geometries of DPPT‐8H, 2DPPT‐1Zn‐14H, 2DPPT‐2Zn‐12H, 2DPPT‐3Zn‐10H, 2DPPT‐4Zn‐8H.

In addition, the RDG (reduced density gradient) scatter plots were calculated to unveil weak interaction differences between PTO, DAP and DPPT. The RDG plots can be divided into three regions (The red region shows strong mutual repulsion, such as steric hindrance and green region means van der Waals effect, such as π–π stacking and blue region means strong attraction, such as hydrogen or halogen bond) [39, 40]. The obvious areal distribution of the green region of DPPT compared to PTO and DAP (Figure 3d and Figures S32 and S33) demonstrated enhanced π–π stacking between DPPT molecules. Moreover, there were more green areas around DPPT molecules than PTO and DAP in visible RDG map (Figure S34), indicating weak interaction (π–π stacking) was stronger than PTO and DAP. The enhanced π–π stacking interaction resulted in more stability of DPPT and long cycling life (45 000 cycles) of DPPT ZOBs (Figure 2e). Besides, there was a narrower band gap (0.560 eV) in DPPT than DAP (0.686 eV) and PTO (1.073 eV), indicating better electrical conductivity for fast electron transfer (Figure 3e and Figures S35 and S36). The electron density at the Fermi level was mostly contributed to *p* orbitals from C═N groups and aromatic rings, verifying that C═N was in favor of facilitating electronic transition.

Moreover, the optimized configuration of the discharged process was calculated. There were 16 discharged products totally and 2DPPT‐1Zn‐14H, 2DPPT‐2Zn‐12H, 2DPPT‐3Zn‐10H, all possessed different isomers (Figure S37). The first isomer of 2DPPT‐1Zn‐14H, 2DPPT‐2Zn‐12H, 2DPPT‐3Zn‐10H showed the lowest G and H values than others, meaning the optimal structure of each Zinc‐contained compositions (Figures S38–S42). Then, they were chosen to be compared in a histogram, as well as DPPT‐8H and 2DPPT‐4Zn‐8H (Figure 3f). 2DPPT‐1Zn‐14H, two DPPT molecules binding with one Zn^2+^ and fourteen H^+^, was the most likely probably configuration in consideration of the concentration of Zn^2+^ is about 3.5×10^4^ times much higher than H^+^ in 3.5 M Zn(ClO_4_)_2_ electrolyte, though DPPT‐8H showed the lowest ΔG and ΔH. To evaluate the water solubility of discharged products, the Log*P* was computed. The DPPT‐8H showed higher value (−2.61) than each optimal structure of four Zinc‐contained compositions because of the existence of zinc‐ionic bonds in Zinc‐contained compositions (Figure S43). Meanwhile, 2DPPT‐1Zn‐14H and 2DPPT‐2Zn‐12H possessed higher Log*P* values than 2DPPT‐3Zn‐10H and 2DPPT‐4Zn‐8H, demonstrating better hydrophobic nature.

### 2.4 The Storage Mechanism of DPPT ZOBs During Charge/Discharge Process

According to literatures, the proton will bind with C═N when discharged [41, 42, 43], so CV curves in 3.5 M Zn(ClO_4_)_2_ (pH = 4) and HClO_4_ (pH = 1) were performed (Figure S44). There all showed a pair of peaks and the orange dotted line in HClO_4_ (pH = 4, shifted by Nerst equation) overlapped with the pink line in 3.5 M Zn(ClO_4_)_2_, proving reaction of proton and Zn^2+^/H^+^ synergistic mechanism. After H^+^ reacted with C═N, OH^−^ would react with Zn(ClO_4_)_2_, forming Zn_4_ClO_4_(OH)_7_ to keep electrical neutrality. Ex situ XRD patterns were performed to prove it (Figure 4a). When it was discharged, the peak at 10.6° of Zn_4_ClO_4_(OH)_7_ (PDF:41‐0715) was enhanced, proving reaction between OH^−^ and Zn(ClO_4_)_2_. Additionally, it was reversible when it was charged, indicating dis‐embedding of H^+^. Besides, the phase around 43°, contributed to Zn(ClO_4_)_2_·6H_2_O (PDF:33‐1470), DPPT and Ketjen Black, showed the opposite behavior, indicating the insertion of proton during discharged progress and de‐intercalation of proton during charged progress. Then, in situ Raman was used to reveal the active center of C═N (Figure 4b). The peak of C═N at 1525 cm^−1^ was gradually weakened during discharged progress, indicating reaction of Zn^2+^ and H^+^ with C═N. Then the peak of C═N was recovered during charged progress, verifying extraction of Zn^2+^ and H^+^. Besides, there was the same phenomenon in Ex situ FT‐IR spectra (Figure S45), revealing reaction of C═N with Zn^2+^ and H^+^ during discharging progress, as well as extraction of Zn^2+^ and H^+^ during charging process. Moreover, the impedance of DPPT ZOBs grew smaller when it was discharged from 1.1 to 0.1 V and it still decreased as charged from 0.1 to 1.6 V, which indicated better electrical conductivity during the activation process (Figure 4c). Meanwhile, there was a strong peak when fully discharged and a weak peak when fully charged in Zn 2p XPS spectra (Figure 4d), meaning embedding of Zn^2+^ during discharging and dis‐embedding of Zn^2+^ during charging. It showed a strong peak of C─N and a weak peak of C═N at full discharged state in XPS N 1s spectra (Figure 4e), confirming the interaction of C═N with Zn^2+^ and H^+^ at discharged. Furthermore, during the process from DPPT to DPPT‐8H, N in C═N showed more obvious electrophilic reactivity, proving that C═N was easy to obtain electrons and reactive center of C═N during the discharge process (Figure 4g, Figures S46 and S47). Additionally, the C atoms in the pyrene backbone of DPPT‐8H exhibited a high nucleophilic reactivity, rendering that water was slightly prone to bind with DPPT‐8H and the stability of DPPT‐8H in water was worse than that of DPPT, which was consistent with the log*P* calculation (Figure S43). Furthermore, The SEM in the different states at the first, 10000^th^, and 45 000^th^ cycles of DPPT ZOBs had been carried out. There were amount of micro or nano flakes in discharged status, which were agreed well with the typical morphology of Zn_4_ClO_4_(OH)_7_. Remarkably, Zn_4_ClO_4_(OH)_7_ gradually disappeared after recharging, proving that the precipitation of Zn_4_ClO_4_(OH)_7_ was highly reversible (Figure S48). Besides, the micro or nano flakes appeared at 0.1 V and disappeared at 1.6 V at the 10 000^th^ and 45 000^th^ cycles, indicating the high reversibility of Zn_4_ClO_4_(OH)_7_ after long cycling and alkaline zinc perchlorate would not permanently contaminate the electrode surface (Figure S49).

**FIGURE 4:**
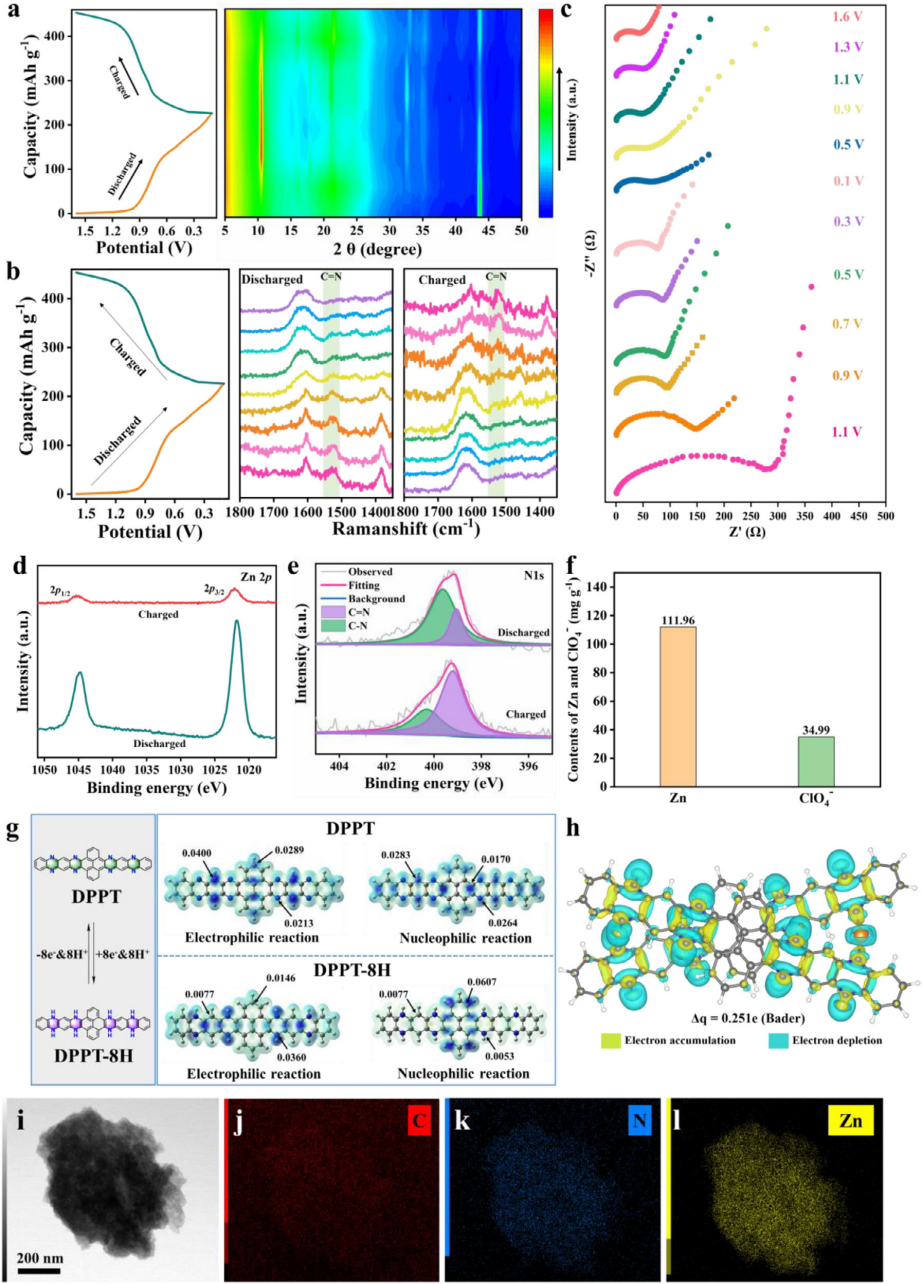
Energy storage mechanism of DPPT ZOBs. (a) Ex situ XRD patterns of DPPT cathodes at different potentials. (b) In situ Raman spectra of DPPT ZOBs. (c) In situ EIS spectra of DPPT ZOBs. (d, e) Ex situ Zn2p and N1s spectra of DPPT cathodes. (f) The contents of Zn by ICP (Inductively coupled plasma) atomic emission spectroscopy and ClO_4_^−^ by IC (Ion chromatography) at fully discharged status. (g) Condensed Fukui Function (CFF) of DPPT and DPPT‐8H. (h) Charge density differences of optimal structure of 2DPPT‐1Zn‐14H. (i‐l) STEM mapping of DPPT cathode at fully discharged state after 45 000 cycles.

Besides, capacity contribution was also calculated (Figure 4f). The contents of Zn by ICP (Inductively coupled plasma) atomic emission spectroscopy at discharged were 111.96 mg g^−1^, while ClO_4_^−^ by IC (Ion chromatography) at discharged were 34.99 mg g^−1^. Thus, weight contents of Zn were 11.196 wt.% and ClO_4_^−^ were 3.499 wt.%, respectively. According to the reaction,

$$4Zn^{2+}+{\;\mathrm{ClO}}_{4}^{-}+7OH^{-}=\;Zn_{4}\mathrm{Cl}O_{4}{\left(\mathrm{OH}\right)}_{7}$$

The molar ratio of H^+^ and Zn^2+^ was

$$\left(7\times \frac{3.499}{99.45}\right):\left(\frac{11.196}{65.39}-4\times \frac{3.499}{99.45}\right)\;=\;0.24626\;:0.03048$$

Thus, the ratio of capacity contribution of H^+^ and Zn^2+^ was

$$\left(7\times \frac{3.499}{99.45}\right):2\times \left(\frac{11.196}{65.39}-4\times \frac{3.499}{99.45}\right)=0.24626\;:0.06096$$

Hence, the capacity contribution of H^+^ was about 80.2% and Zn^2+^ was around 19.8%.

To research the morphology of DPPT after 45 000 cycles, STEM mapping of DPPT ZOBs after 45 000 cycles was studied (Figure 4i‐l). DPPT still showed the morphology of nanoflake after long cycles of 45 000, indicating the stability of DPPT for extended π‐conjugation, enhanced π‐π interaction and good aromaticity. Moreover, the Zn element was evenly distributed in DPPT, verifying insertion of Zn^2+^ at fully discharged state. After zinc‐ion and proton insertion, there was an obvious charge transfer between zinc‐ion & protons and C═N sites in optimal structure of 2DPPT‐1Zn‐14H (Δq = 0.251e), resulting in a stable ligand configuration (Figure 4h).

### 2.5 Characterizations of Flexible DPPT ZOBs and Application in Wearable NH_3_ Sensors

Presently, most reported organic cathode materials of ZOBs, such as MBTS showed low stability below 10 000 cycles (Figure 5a, green region). A few materials, such as 3TANC could only reach 10 000 cycles, yet below 20 000 cycles (blue region) and seldom materials, such as DQH showed high stability beyond 20 000 cycles (red region) [21, 23, 24, 25, 40, 41, 42, 44, 45, 46, 47, 48, 49, 50, 51, 52, 53, 54, 55, 56, 57, 58, 59, 60, 61, 62, 63, 64, 65, 66, 67, 68, 69, 70, 71, 72, 73, 74, 75, 76, 77, 78, 79]. In this work, DPPT ZOBs showed ultrahigh stability of 45 000 cycles with 83% capacity retention, owning to more electrons distributed in DPPT molecule, enhanced π‐π stacking of DPPT molecules and good aromaticity. Based on the good electrochemical performance of DPPT, flexible DPPT ZOBs were fabricated by Zn anode, Zn(ClO_4_)_2_ hydrogel electrolyte [80], DPPT cathode (Figure 5b).

**FIGURE 5:**
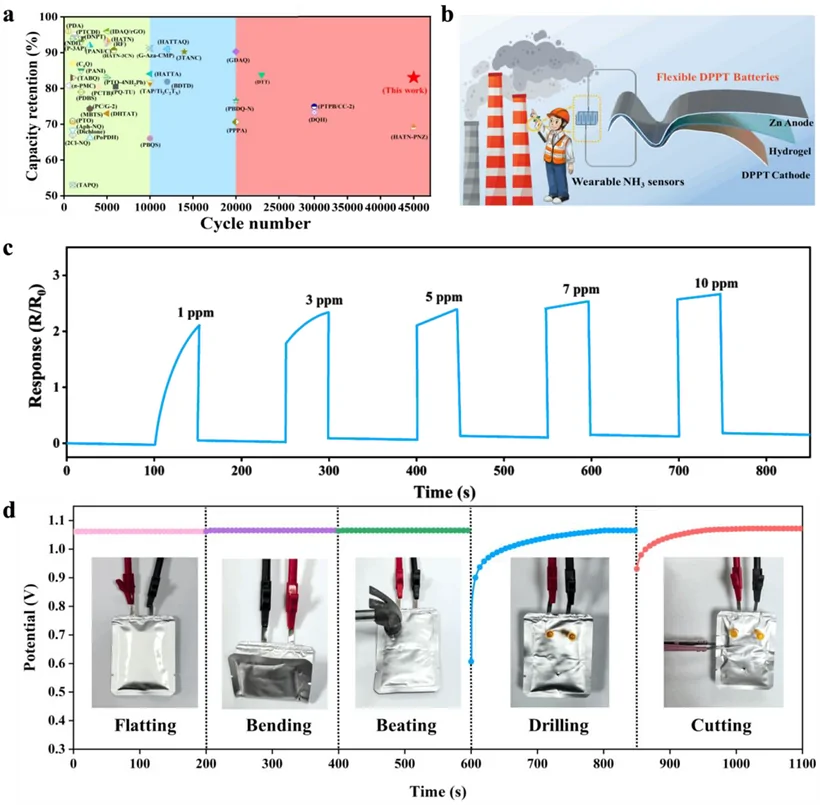
Application of flexible ZOBs for wearable NH_3_ sensors. (a) Stability comparison of DPPT with reported ZOBs cathodes. (b) Schematic diagram of flexible DPPT ZOBs supplying energy for wearable NH_3_ sensors. (c) Response curve of NH_3_ sensors powered by flexible DPPT ZOBs. (d) Potential‐time curves of flexible DPPT ZOBs at states of flatting, bending, beating, drilling and cutting.

In order to develop wearable power supply, flexible DPPT ZOBs were used to supply energy for wearable NH_3_ sensors (Figure 5b), which could help real‐time monitoring NH_3_ concentration in surrounding environment. As a result, wearable NH_3_ sensors supplied energy by flexible DPPT ZOBs showed obvious responses with increasing NH_3_ concentration from 1 to 10 ppm (Figure 5c). Moreover, there was good linear correlation of response in different NH_3_ concentration (Figure S50), verifying good ammonia sensing performance for further application.

DPPT ZOBs also demonstrated stable potential of 1.06 V at flatting, bending, and beating states, verifying good flexible performance and anti‐strike ability (Figure 5d). Impressively of being drilled and cut, the potential would recover into 1.06 V in 200s, indicating anti‐drilling and cuttability of flexible DPPT ZOBs. Besides, drilling and cutting could not lead to a complete short circuit because it couldn't make the contact between the cathode and anode without forming a complete internal conductive loop. The voltage recovered spontaneously after the initial drop because the drilling was reversible soft breakdown rather than permanent hard short‐circuiting and the Zn(ClO_4_)_2_ hydrogel electrolyte possessed good elasticity and self‐healing ability, which could elastically rebound and automatically close tiny puncture or breakdown pores to re‐isolate the cathodes and anodes and cut off transient conductive paths. Meanwhile, the instantaneous short‐circuit current disappeared, and electrochemical polarization as well as concentration polarization inside the battery gradually relaxed and dissipated. With the internal polarization drop vanishing, the open‐circuit voltage returned to its normal level. In addition, the flexible DPPT ZOBs possessed structural fault tolerance, so local damage would not cause overall failure, enabling the battery voltage to restore significantly after breakdown. Ultimately, flexible DPPT ZOBs still could lighten a LED lamp after being bent, beaten, drilled and cut (Figure S51). Prospectively, wearable NH_3_ sensors supplied energy by flexible DPPT ZOBs would promote industrial application from as‐fabricated Zinc‐organic batteries.

## 3 Conclusions

In this work, original C═N material of DPPT with eight C═N active sites and enhanced π‐π stacking has been designed for Zinc‐organic batteries (ZOBs), showing long cycling life of 45 000 cycles with 83% capacity retention at 5 A g^−1^ and good rate performance of 20C. Notably, the capacity is increased from 110 to 185 mAh g^−1^ after 400 cycles at 5 A g^−1^, indicating obvious activation process. The conversion from DPPT‐8H (formed by the reaction of DPPT with eight protons during discharged process) to DPPT is the key reason for activation process. Through Ex situ ^1^H‐NMR and in situ Raman, the transformation of DPPT‐8H into DPPT is proved, resulting in more active sites and increased capacity. Besides, the active sites of C═N in DPPT and synergetic mechanism of Zn^2+^ & H^+^ are confirmed by in situ experiments. After DFT calculation, the most likely configuration is 2DPPT‐1Zn‐14H forming by two DPPT molecules with one zinc ion & fourteen protons after calculating 16 possible discharging products. Impressively, flexible DPPT ZOBs with sandwich structure are assembled and can work at flatting, bending, beating, drilling and cutting states, indicating anti‐strike ability, anti‐drilling ability and cuttability. In addition, flexible DPPT ZOBs can supply energy for wearable NH_3_ sensors, demonstrating good ammonia sensing performance. The DPPT ZOBs with extraordinary cycling life and high capacity would stimulate the development of ZOBs and promote applications in wearable devices.

## 4 Experimental Section

### 4.1 Synthesis of DPPT

DPPT was synthesized by PTO (Pyrenetetraone) and DAP (Phenazine) through one step reaction. First, DAP (7 mmol) and PTO (3.5 mmol) were dissolved in acetic acid (35 mL) and ethanol (35 mL) solution in a Schlenk tube. Then it was heated under refluxing at 140°C for 24 h under the protection of N_2_. For purifying, the suspension was filtered and boiled with 30 wt.% HNO_3_ at 140°C for 3 h and repeat the process for three times then filtered and washed by deionized water, chloroform, ethanol for several times. Finally, the reddish‐brown DPPT powder was obtain after being dried in a vacuum oven at 120°C for 12 h.

## Author Contributions

**Xiaodong Geng**: conceptualization, methodology, software, data curation, formal analysis, validation, investigation, writing – original draft, writing – review and editing, visualization. **Zhangyu Wang**: data curation. **Qian Zhao**: data curation. **Weiyao Bi**: data curation. **Kai Yang**: data curation. **Hongting Ma**: data curation. **Nan Zhu**: writing – original draft, writing – review and editing, conceptualization, funding acquisition, project administration, resources. **Qinghua Deng**: data curation. **Zhiqian Li**: data curation. **Ying Liu**: data curation. **Yuqian Jiang**: theoretical calculation, writing – original draft, writing – review and editing. **Dan Zhao**: writing – original draft, writing – review and editing.

## Conflicts of Interest

The authors declare no conflicts of interest.

## Supporting information




**Supporting File 1**: advs76326‐sup‐0001‐SuppMat.docx.

**Supporting File 2**: advs76326‐sup‐0002‐VideoS1.mp4.

## Data Availability

The data that supports the findings of this study are available in the supplementary material of this article.
