# Supplementary material for: Unveiling Activation Process of C═N Cathode for High‐Performing Zinc‐Organic Batteries
Source: Adv Sci (Weinh). 2026 Jun 28:e76326. Online ahead of print. doi: 10.1002/advs.76326 (PMC13336899; doi:10.1002/advs.76326)
Supplement: Supplementary file 1 — Supporting File 1: advs76326‐sup‐0001‐SuppMat.docx. [file ADVS-9999-e76326-s002.docx]

Copyright WILEY-VCH Verlag GmbH & Co. KGaA, 69469 Weinheim, Germany, 2026.

Supporting Information

Unveiling Activation Process of C=N Cathode for High-Performing Zinc-Organic Batteries

Xiaodong Geng^1^, Zhangyu Wang^1^, Weiyao Bi^1^, Qian Zhao^1^, Hongting Ma^1^, Kai Yang^1^, Ying Liu^1^, Zhiqian Li^1^, Qinghua Deng^1^, Dan Zhao^3^*, Yuqian Jiang^2^*, and Nan Zhu^1^*

1. Experimental Section

*Electrochemical measurements:* The coin cells (CR2032) and typical three-electrode systems were assembled with a mass loading of DPPT cathode was about 1-2 mg cm^-2^ for electrochemical measurements. Coin-type batteries were assembled with DPPT cathode, Zn anode (thickness was 0.2 mm with a diameter of 16 mm), double glass fiber (Whatman GF/D+ GF/C) as separator, and 3.5 M Zn(ClO_4_)_2_ as electrolyte, tested on the Neware test system at 0.1-1.6 V. In addition, CV curves of DPPT electrode in HClO_4_ (pH=1) electrolyte and 3.5 M Zn(ClO_4_)_2_ electrolyte were measured by three-electrode system (DPPT electrode as working electrode, Platinum as counter electrode, Ag/AgCl as reference electrode).The Cyclic voltammetry (CV) & Electrochemical impedance spectra (EIS) were performed on the PGSTAT302N potentiostat/galvanostat (Metrohm, Autolab B.V., Utrecht, The Netherlands; serial no.: AUT88992). Besides, Zn(ClO_4_)_2_-polyacrylamide/chitosan hydrogel electrolyte (C-PAMCS) was prepared for flexible DPPT Zinc-organic batteries. In addition, ion diffusion coefficients (D) using galvanostatic intermittent titration technique (GITT) is determined using following Equation,

$$D=\frac{4}{\pi\tau}\left( \frac{m_{B}V_{M}}{M_{B}S} \right)^{2}\left( \frac{\Delta E_{s}}{\Delta E_{\tau}} \right)^{2}$$

Where m_B_ is the mass of active material in the electrode; τ is the duration of the galvanostatic current pulse; S is the electrode area; V_M_ is the molar volume of active material; M_B_ is the molar mass of the active material; ΔE_τ_ is the voltage difference between beginning and termination of one single step; and ΔE_S_ is the difference in open-circuit voltages after two adjacent relaxation period.

*Fabrication of the NH_3_ sensors:* Firstly, interdigital electrodes, which used Ag/AgCl ink, were fabricated by screen printing. Then, the copper wires were pasted (using commercial silver ink ) to electrodes and heated at 60 °C to dry. Finally, 20 mg mL^-1^ MXene (Ti_3_C_2_T_x_, T means surface terminals) solution was dropped to cover the electrode and NH_3_ sensors were obtained after being dried at 60 °C for 1 h.

*Cleaning process before the ICP/IC test:* According to the test requirements of ICP/IC, each 50 mg electrode materials should be prepared. The cathodes after discharging process were washed by deionized water for many times to remove all soluble Zn(ClO_4_)_2_, then dried in an oven to remain the undissolved basic zinc precipitates. Next, electrode materials were collected to 50 mg by scraping cathodes with a knife.

*Material characterizations:* The morphology of DPPT was measured by Scanning electron microscope (SEM, Hitachi, SU 8220) and transmission electron microscope (TEM, JEM-F200). The characterizations of DPPT and *Ex-situ* characterizations of DPPT cathode were performed by 400 MHz NMR spectrometer (Bruker AV400), high-resolution liquid chromatography-mass spectrometry (LC-MS, Q Exactive Plus ), Fourier transform infrared spectroscopy Spectrometer (FT-IR, IRTracer-100, using ATR), X-ray powder diffractometer (XRD, D/Max 2400), X-ray photoelectron spectroscopy (XPS, ESCLAB 250Xi), transmission electron microscope (TEM, JEM-F200) at room temperature. In addition, *in-situ* experiments were tested on Raman spectrometer (Renishaw) and electrochemical workstation (Atuolab).

*Calculation based on Nernst Equation:*

Electrode reaction:

$$DPPT+8H^{+}+8e^{-}\rightleftharpoons DPPT{(H^{+})}_{8}$$

Nernst Equation:

$\varphi=\varphi^{\theta}+\frac{2.303RT}{nF}lg\frac{\left[ DPPT \right]\left[ H^{+} \right]^{8}}{\left[ DPPT{(H^{+})}_{8} \right]}$= $\varphi^{\theta}+\frac{0.0592}{n}lg\frac{\left[ DPPT \right]\left[ H^{+} \right]^{8}}{\left[ DPPT{(H^{+})}_{8} \right]}$

Where n is electron transfer numbers; F is Faraday constant: 96500 C mol^-1^; R is universe gas constant: 8.314 J K^-1^ mol^-1^; φ is electrode potential; φ^θ^ is standard potential; T is temperature: 298.15 K; the activity of solid DPPT and DPPT(H^+^)_8_ is considered as 1, and n is 8, thus the equation could be further simplified as:

$\varphi=\varphi^{\theta}+\frac{0.0592}{8}lg\left[ H^{+} \right]^{8}$= $\varphi^{\theta}+0.0592lg\left[ H^{+} \right]$

So, the potential difference of DPPT in HClO_4_ solution (pH=1) and 3.5 M Zn(ClO_4_)_2_ solution (pH=4) can be calculated as:

$\Delta\varphi=0.0592 lg\frac{\left[ H^{+} \right]_{1}}{\left[ H^{+} \right]_{2}} V$ $=0.0592 lg\frac{{10}^{-1}}{{10}^{-4}}$V = 0.1776 V

*Calculation of the theoretical capacity of DPPT:* The theoretical capacity of DPPT can be calculated by the following formula:

C_t_ = $\frac{n \times F (C \mathrm{mol}^{-1})}{M_{w} (g \mathrm{mol}^{-1})}$=$\frac{n \times96485 (C )}{M_{w} (g )}$=$\frac{n \times96485 (A s )}{M_{w} (g )}$ = $\frac{n \times96485 \times1000/3600 (mA h )}{M_{w} (g )}$

= $\frac{n \times26801}{M_{w}}$(mAh g^-1^)

Where C_t_ is the theoretical capacity; n is the transferred electron number in each structural unit, F is the Faraday constant, and M_w_ means the molecular weight of the organic molecule. In DPPT, n is 8 and M_w_ is 610.64. Hence, the theoretical capacity of DPPT is 8×26801/610.64 = 351 (mAh g^-1^).

*Calculating the LogP:* Log*P* were calculated by the following formula,

$$\log P=\frac{\Delta Gw-\Delta Go}{RTln10}$$

Where, R is universe gas constant: 8.314 J K^-1^ mol^-1^; T is temperature: 298.15 K;

ΔGw is related to the Gibbs free energy of transfer between water and vacuum phases; ΔGo means the Gibbs free energy of transfer between 1-octanol and vacuum phases;

*DFT calculations:* DFT calculations were computed by the Gaussian 16 program package^[1]^ and all geometry optimizations and frequency analyses were applied by ωB97X-D/6–31+G(d) for the sake of better describing the noncovalent interactions^[2]^. Besides, HOMO-LUMO calculation were using the basis set of b3lyp-D3/def2-SVP. In addition, The Gibbs free energy or enthalpy of each system was obtained for considering the thermal correction from the frequency analysis. Besides, LOL(localized orbital locator)-π color-filled maps, ICSS, RDG (reduced density gradient) scatter plots were calculated by the *Multiwfn*^[3]^. The Band structure and DOS were computed by the Device Studio. Moreover, the GIMIC and ACID were computed by the GIMIC-2.2.0 package and AICD-2.0.0 package.

2. Results and discussion

2.1. Theoretical calculations.


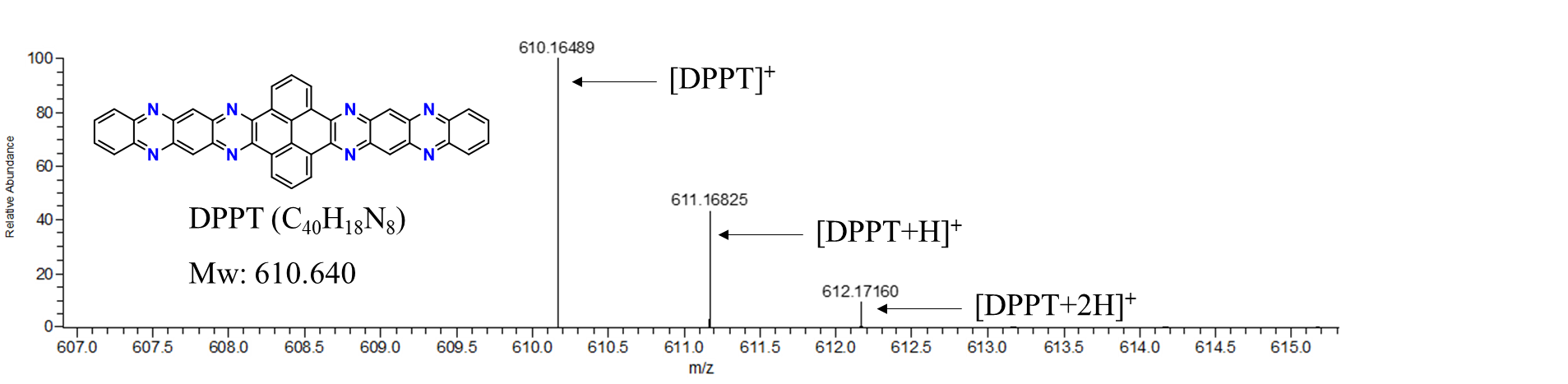


**Figure S1.** Mass spectrum of DPPT.


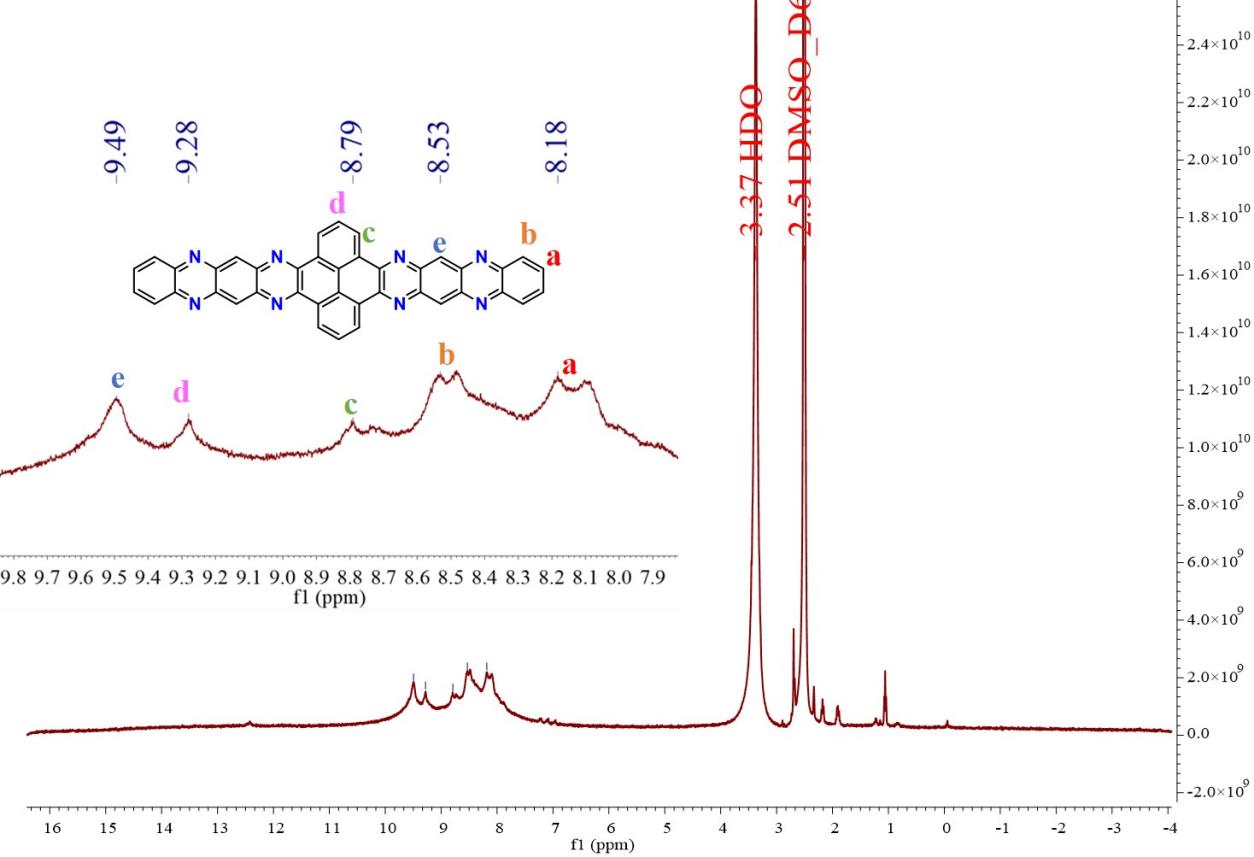


**Figure S2.** ^1^H-NMR spectrum of DPPT





**Figure S3.** FT-IR spectra of DPPT.


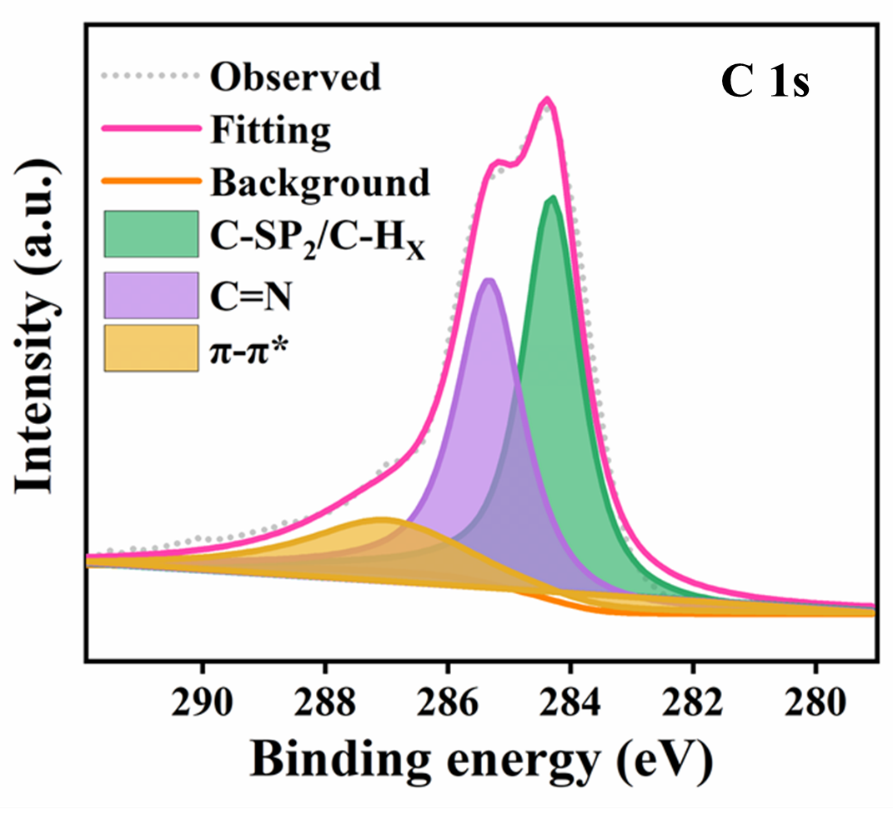


**Figure S4.** C 1s XPS spectra of DPPT.


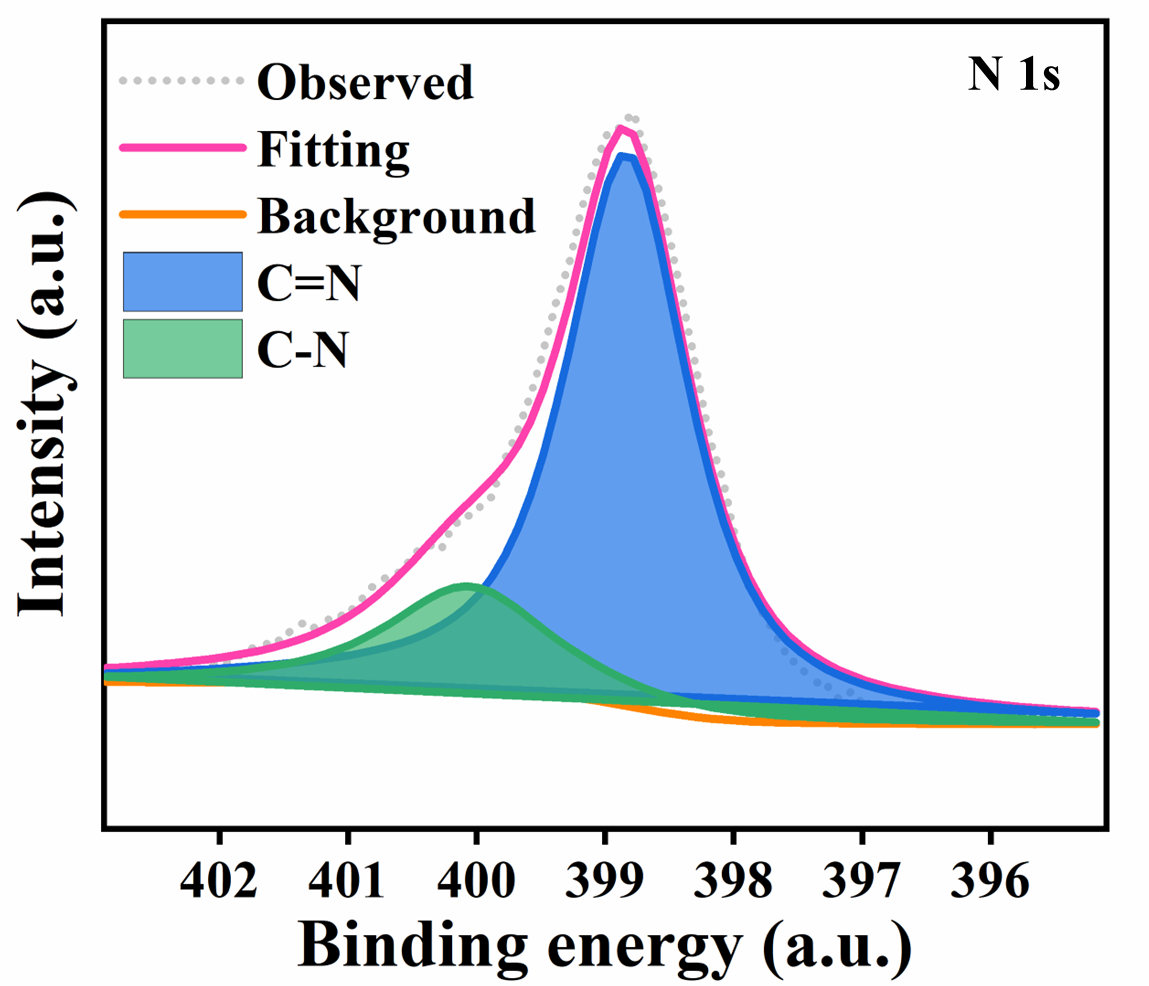


**Figure S5.** N1s XPS spectra of DPPT.


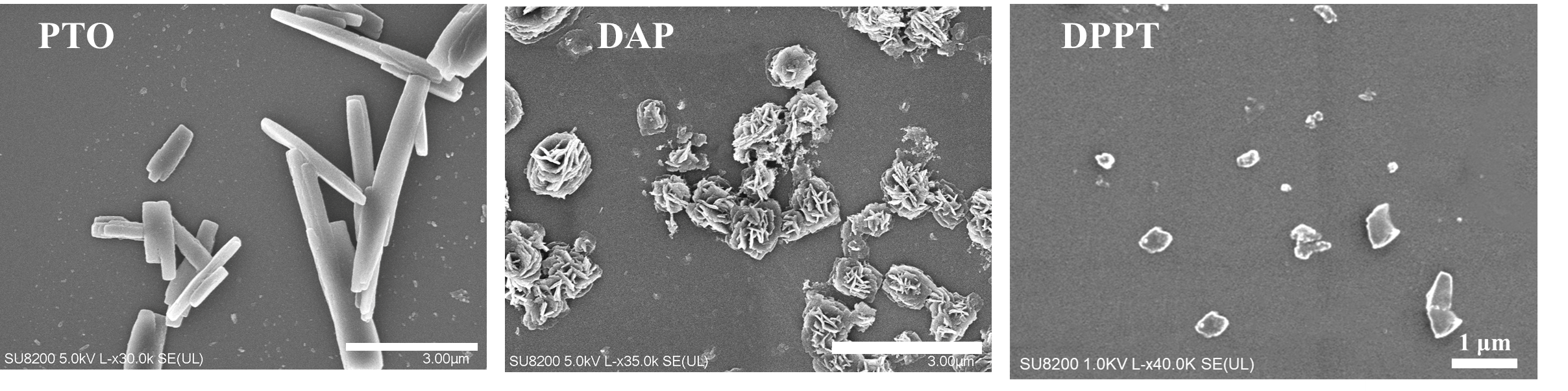


**Figure S6.** SEM images of PTO, DAP and DPPT.


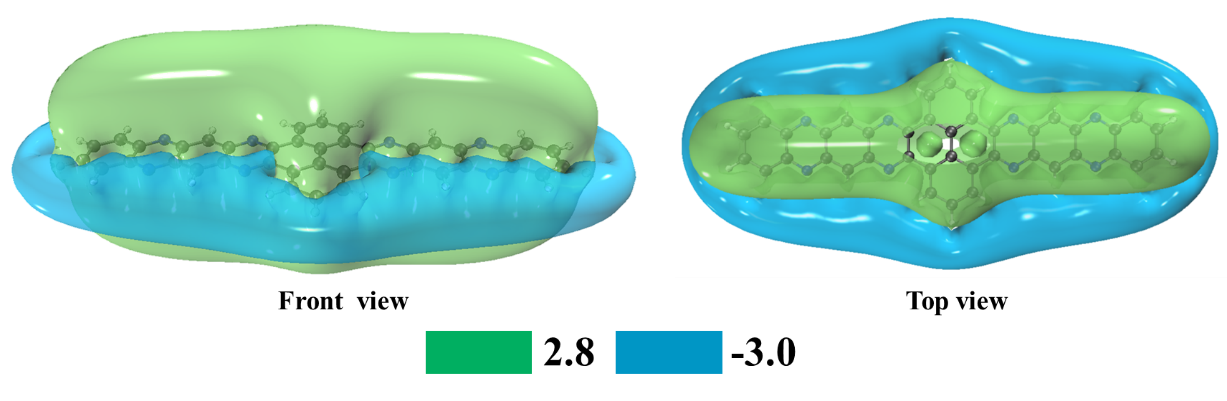


**Figure S7.** Front and top view of ICSS_ZZ_ of DPPT.

**
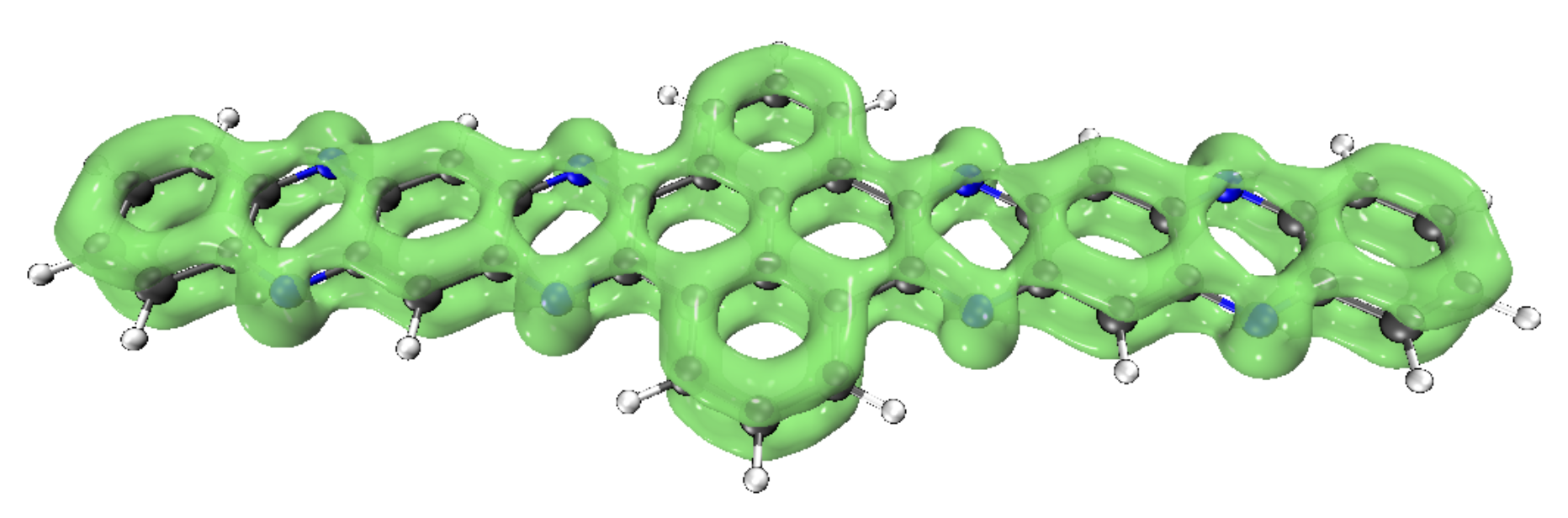
**

**Figure S8.** LOL(localized orbital locator)-π grid map of DPPT.

**
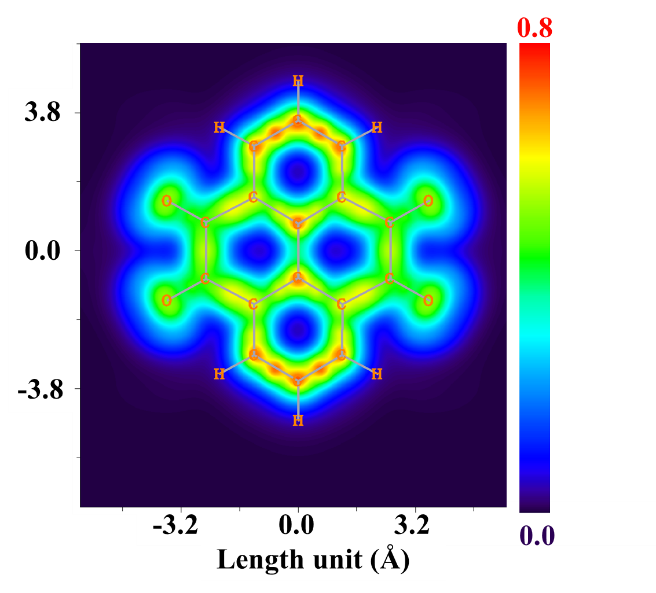
**

**
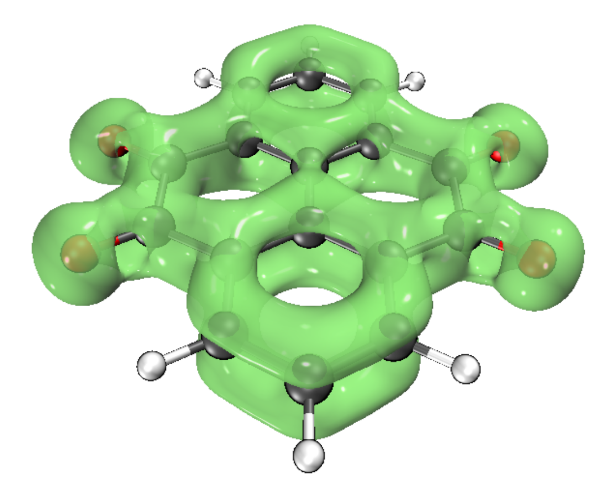
**

**Figure S9.** LOL(localized orbital locator)-π color and grid map of PTO.

**
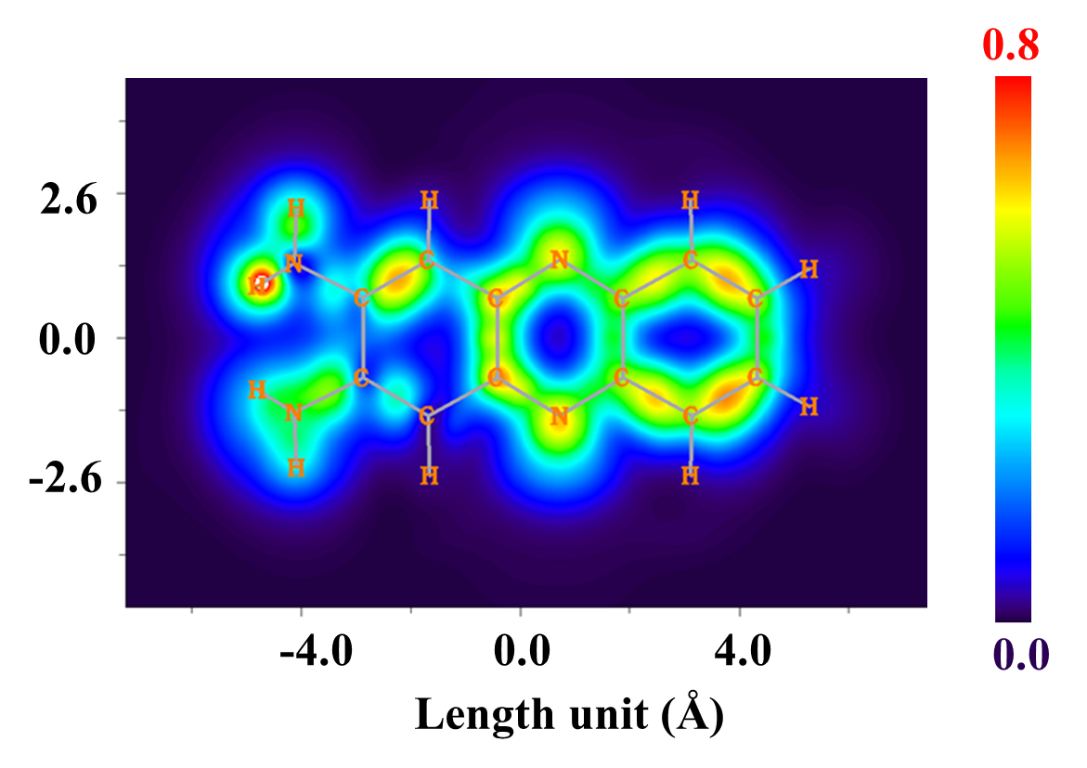
**

**
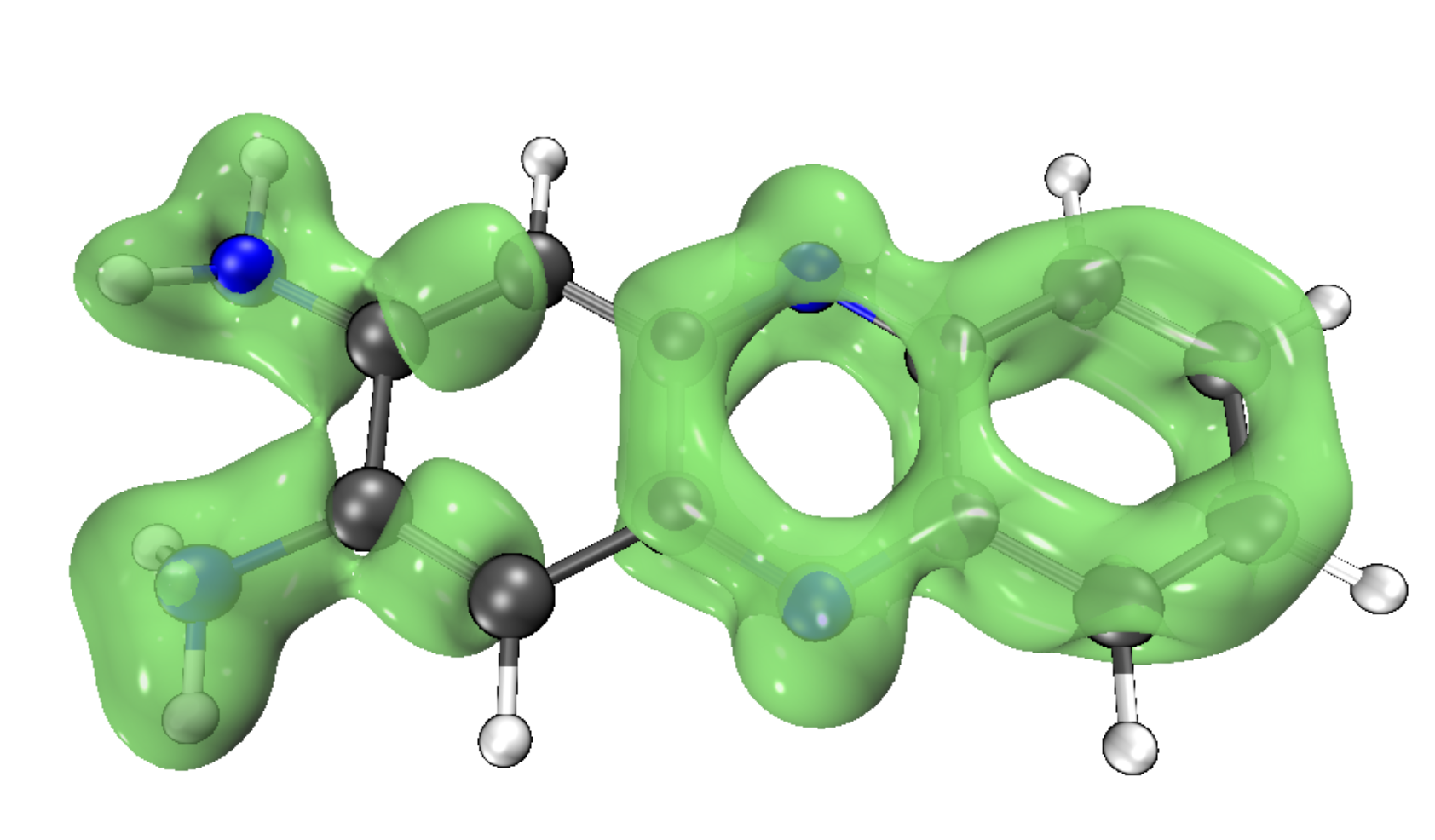
**

**Figure S10.** LOL(localized orbital locator)-π color and grid map of DAP.


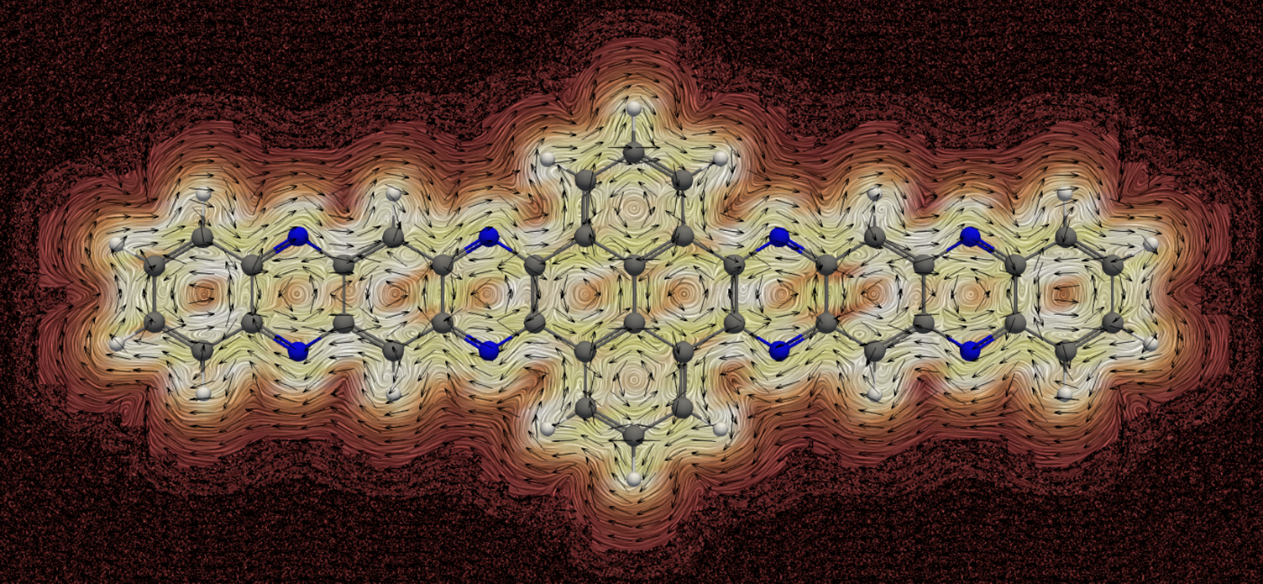


**Figure S11.** GIMIC planar coloring streamlines and arrow diagram of DPPT.

2.2. Activation mechanism and kinetic & electrochemical performance of DPPT ZOBs.


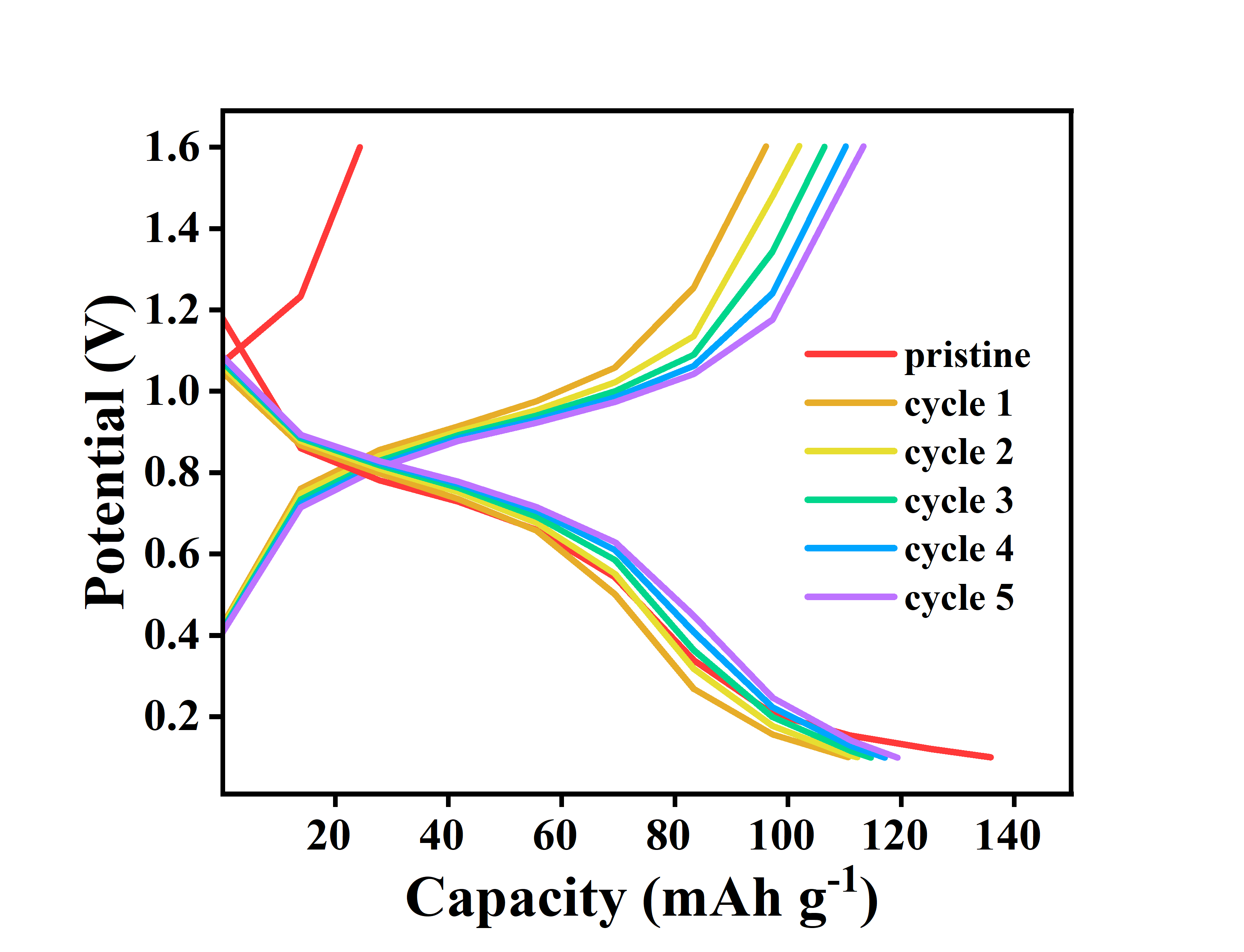


**Figure S12.** GCD curves of DPPT ZOBs at pristine and first 5 cycles.


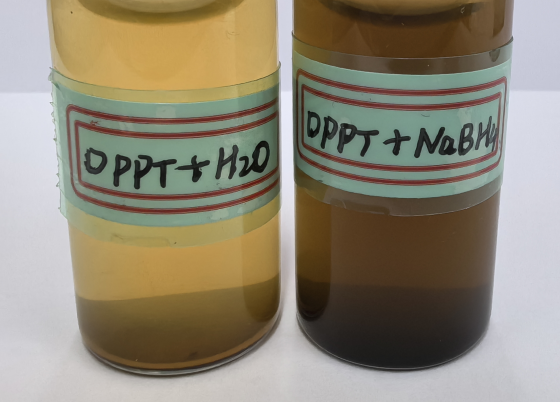

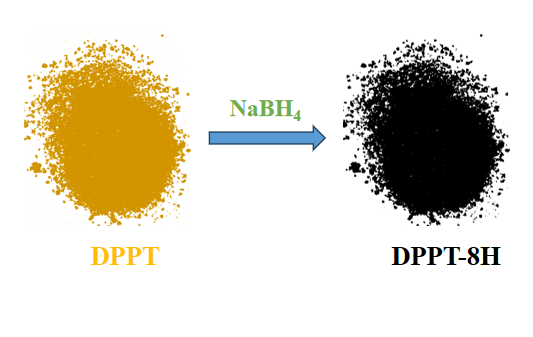


**Figure S13.** Synthetic procedure of DPPT-8H.


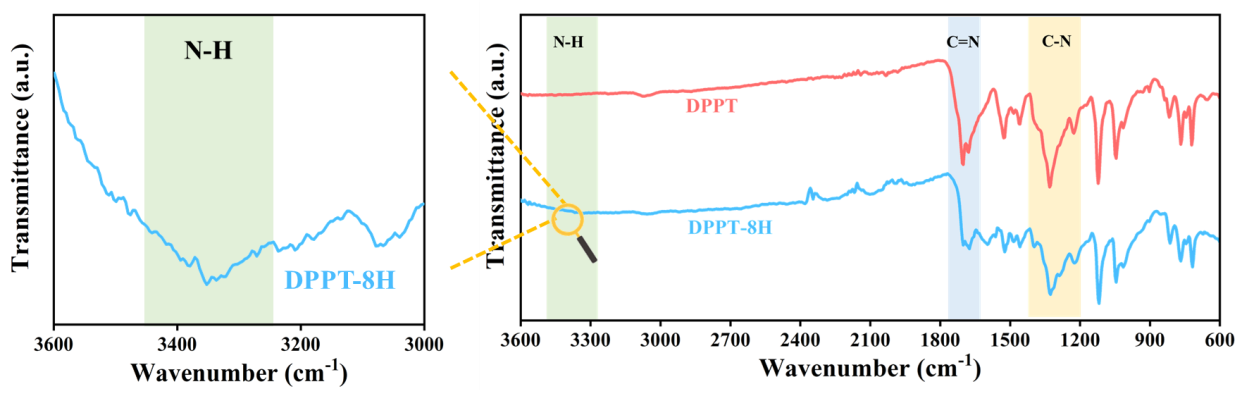


**Figure S14.** FT-IR spectrum of DPPT-8H.

**
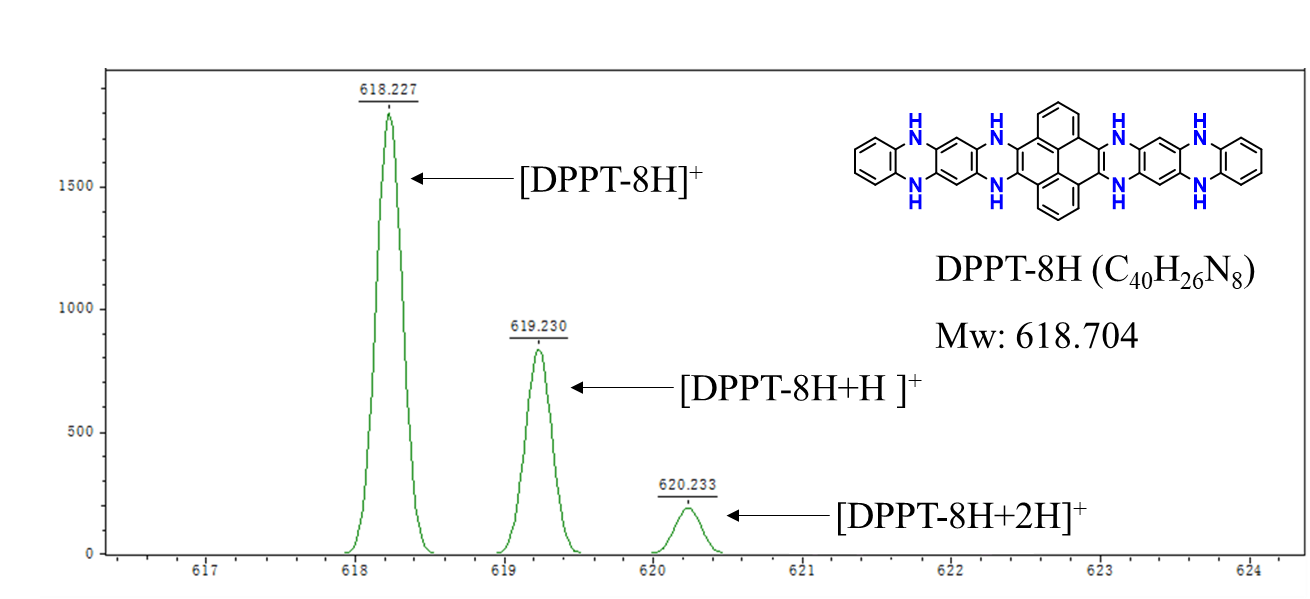
**

**Figure S15.**Mass spectra of DPPT-8H.


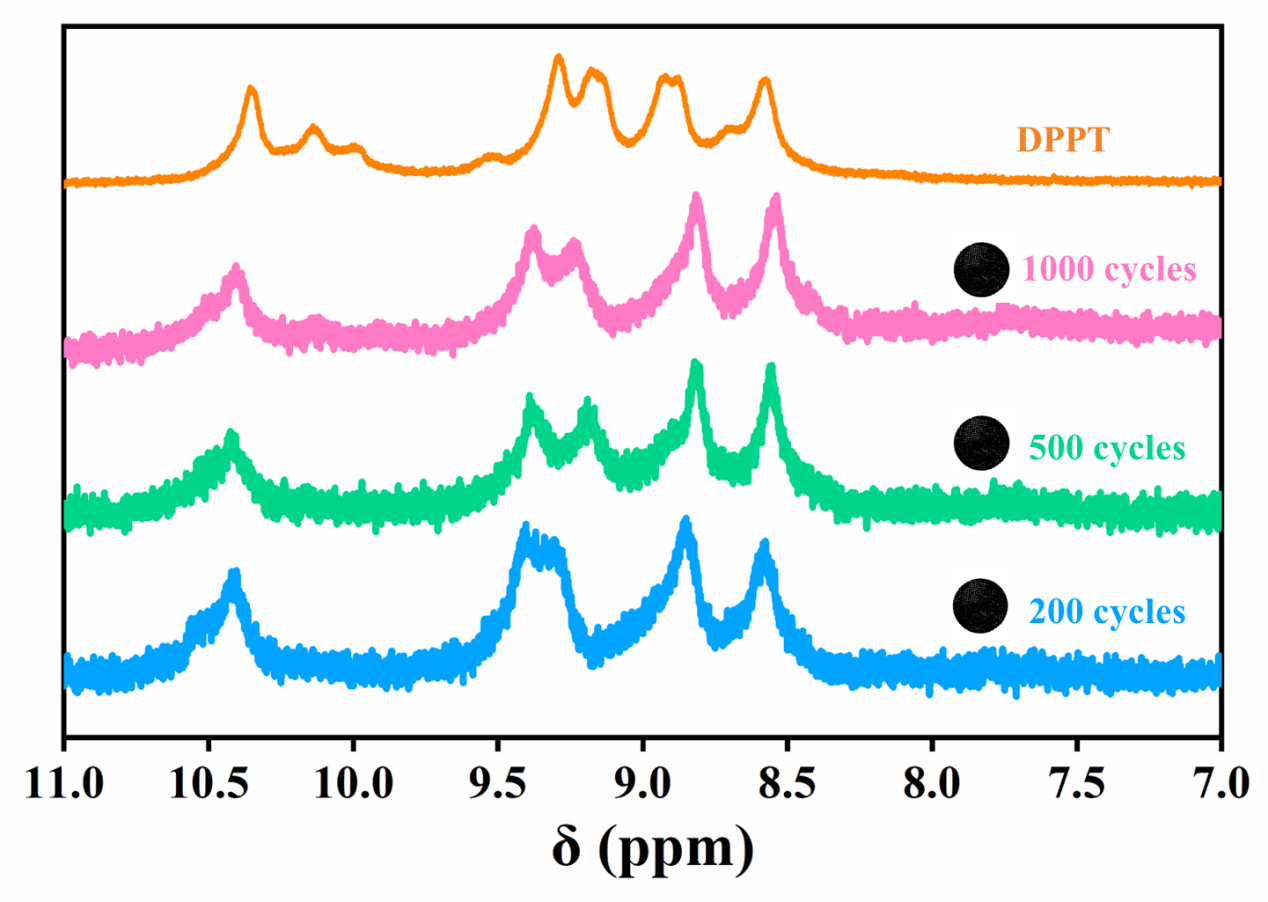


**Figure S16.** *Ex-situ* ^1^H-NMR of DPPT and DPPT-8H cathodes at different cycle numbers.


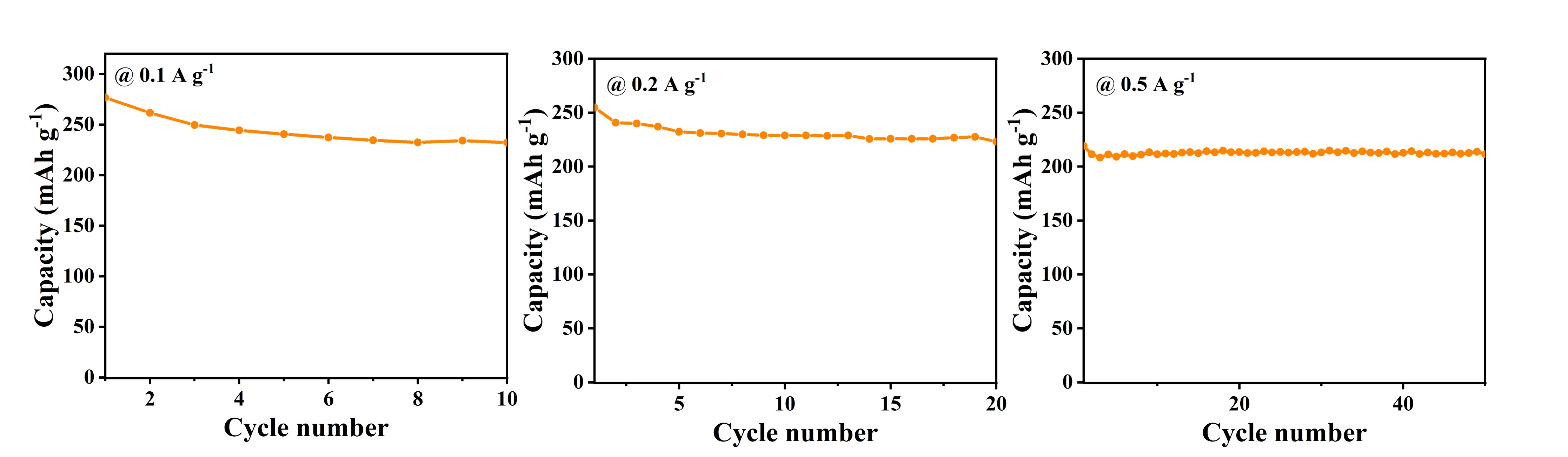
**Figure S17**. The capacity of DPPT ZOBs at the low current densities of 0.1A g^-1^, 0.2A g^-1^ and 0.5A g^-1^.


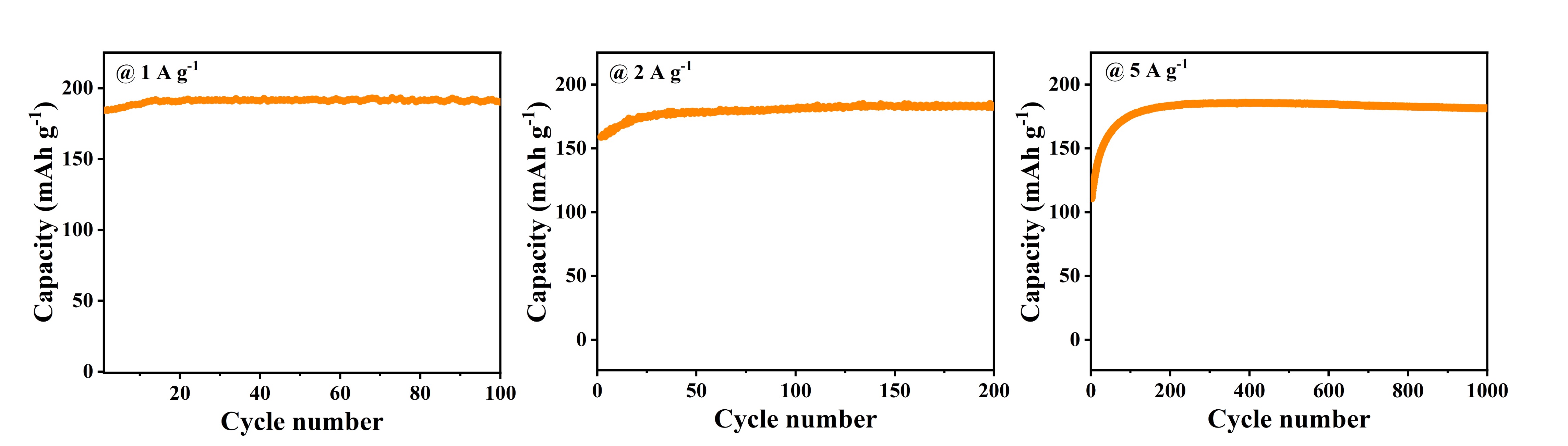


**Figure S18.** The capacity of DPPT ZOBs at high current densities of 1A g^-1^, 2A g^-1^ and 5A g^-1^.


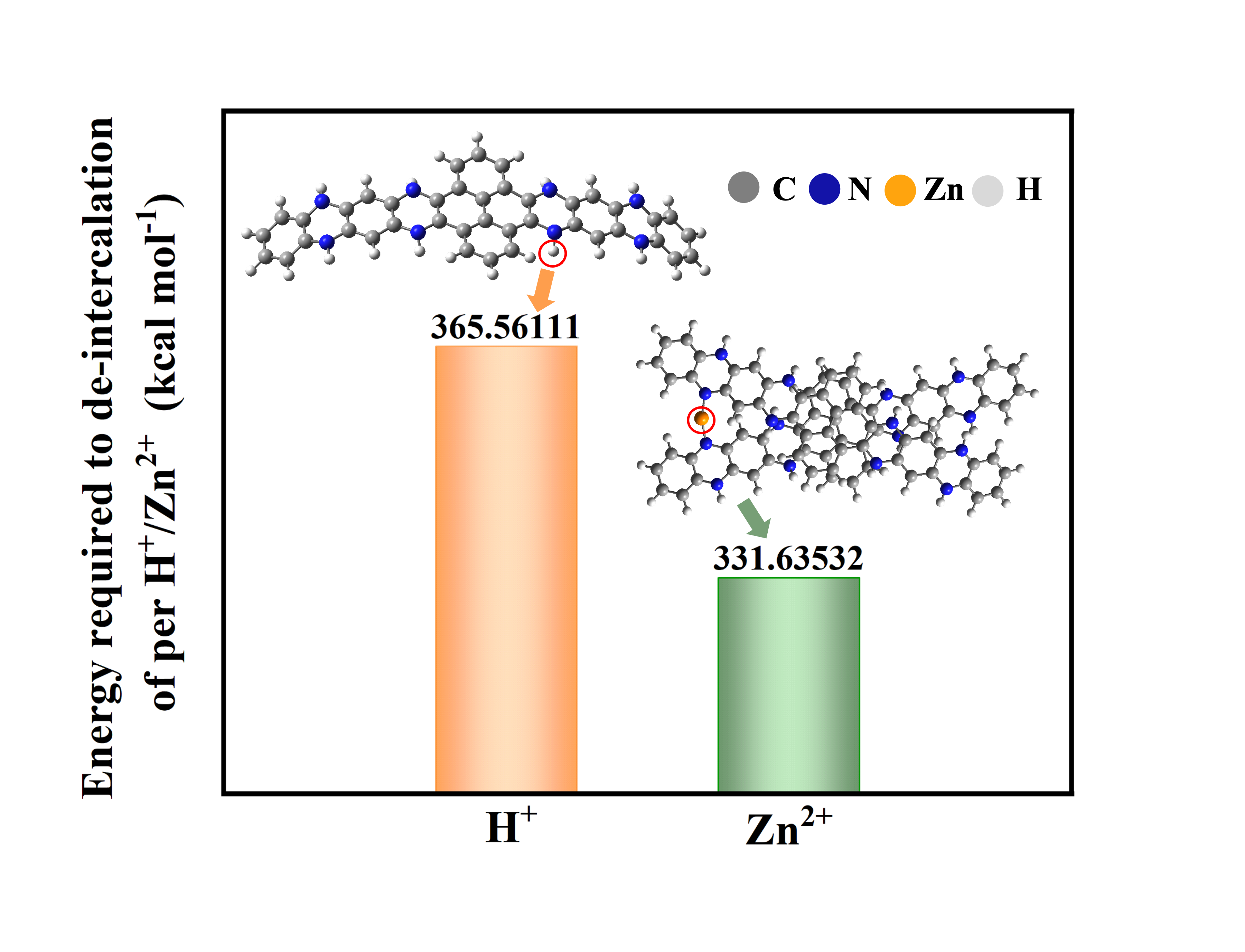


**Figure S19.** The energy required to the de-intercalation of per H^+^ or Zn^2+^.


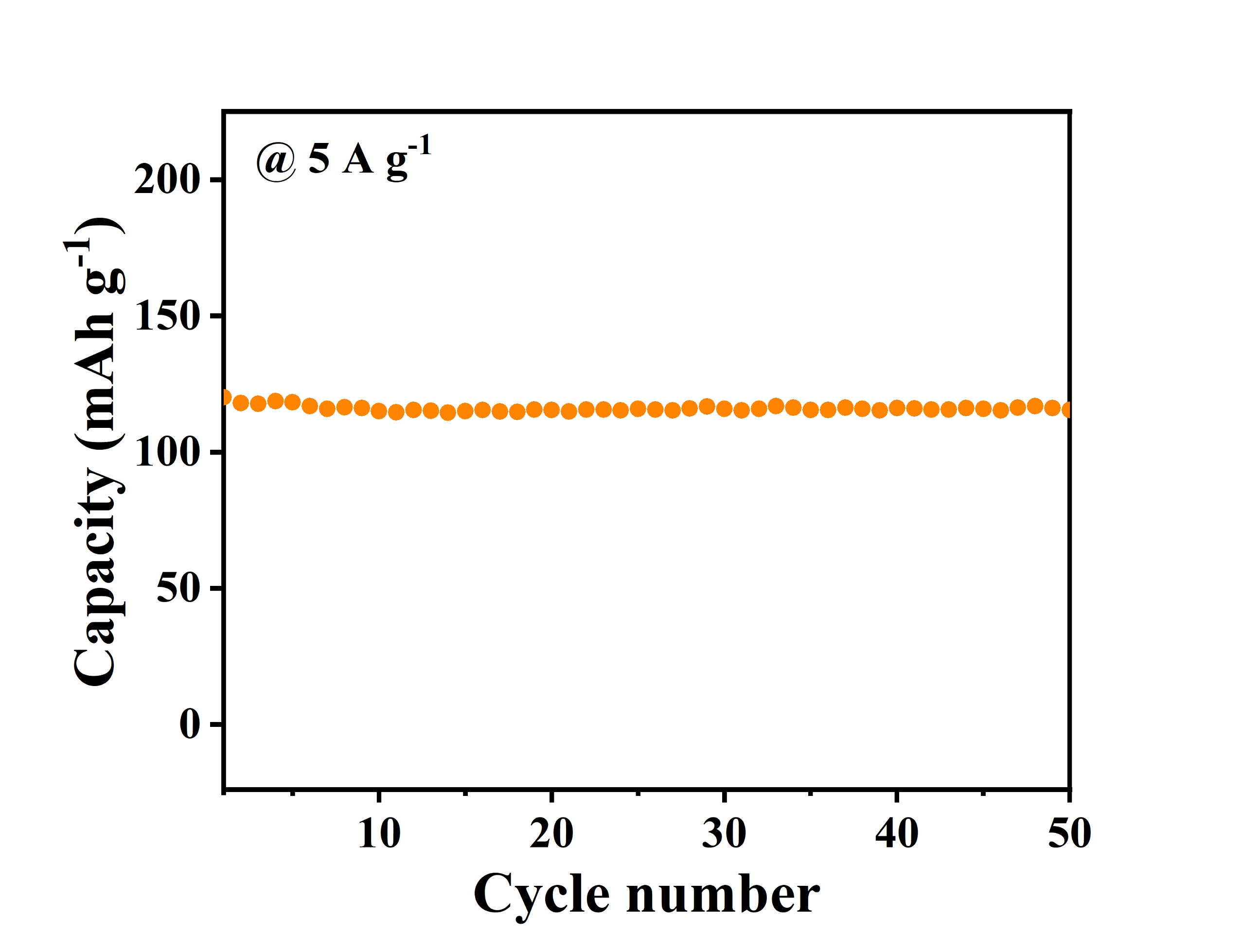


**Figure S20.** The capacity of DPPT ZOBs, using saturated Zn(ClO_4_)_2_/Acetonitrile electrolyte (not containing any H^+^) at 5A g^-1^.





**Figure S21.** CV curves of DPPT Zinc-organic batteries (ZOBs) at 1 mV s^-1^.


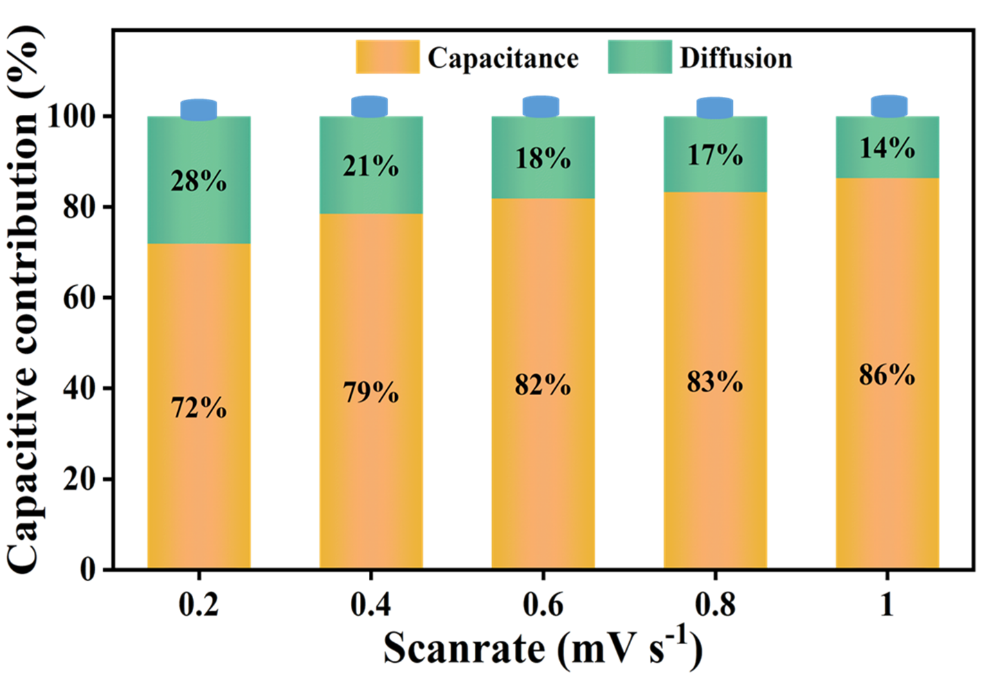


**Figure S22.** Capacitance and diffusion contributions at different scan rates from 0.2 to 1 mV s^-1^.


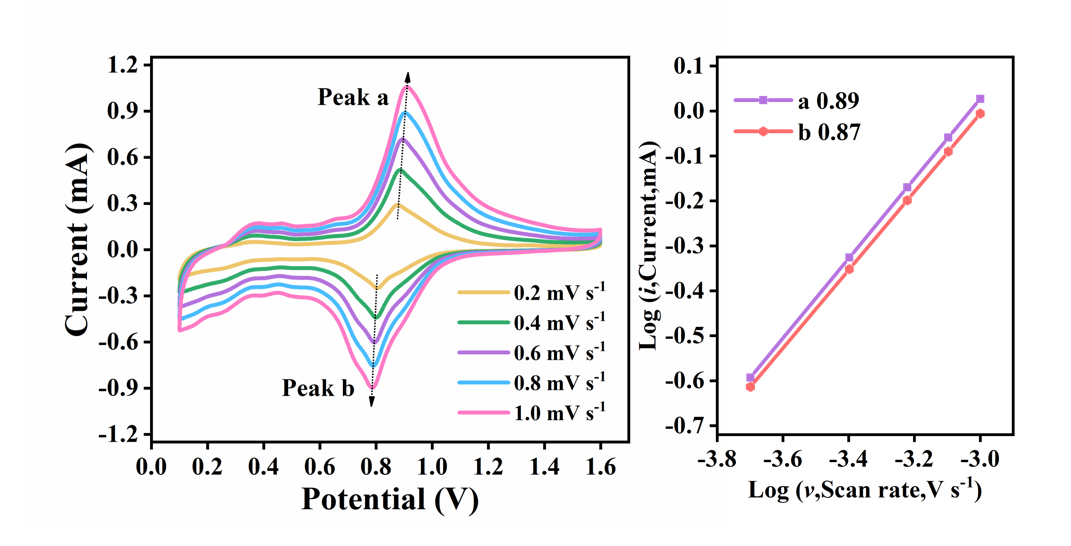


**Figure S23.** CV curves of DPPT at different scan rates and log (*i*) *versus* log(ν) plots of cathodic and anodic current responses at two marked peaks.





**Figure S24.** GITT curve of DPPT ZOBs.





**Figure S25.** Potential-time curve of DPPT ZOBs.





**Figure S26.** Diffusion coefficient (D) calculated by GITT method in discharging progress.


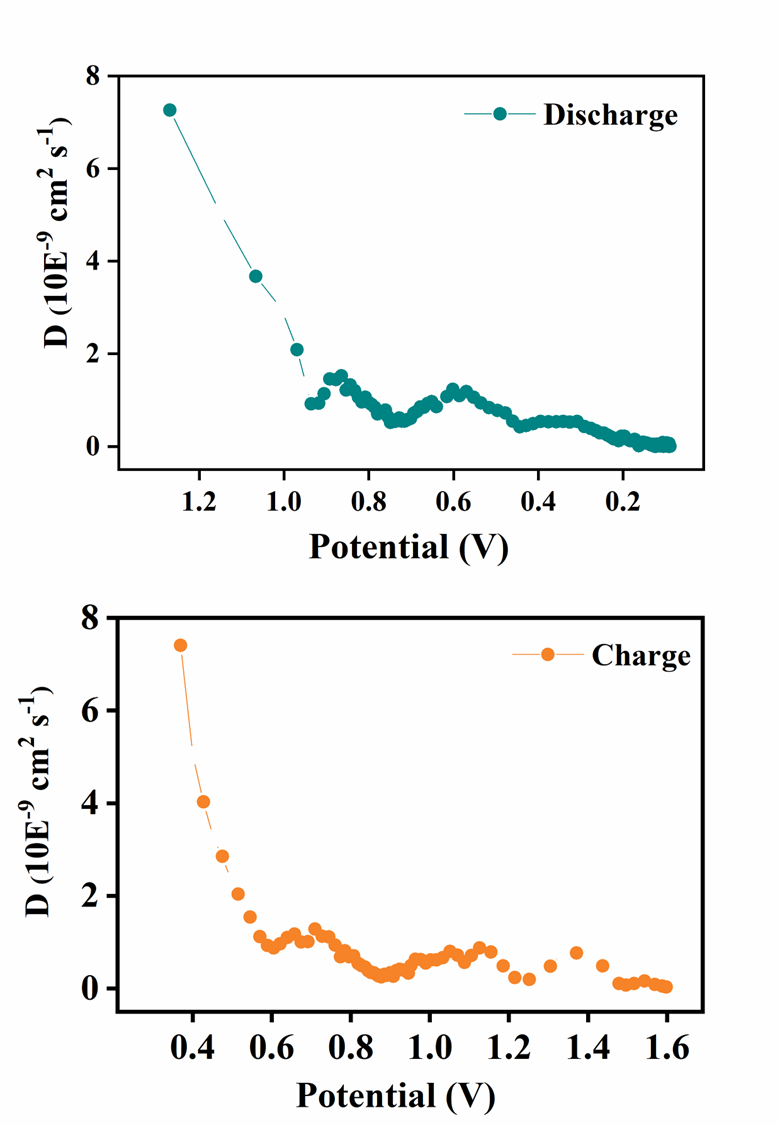


**Figure S27.** Diffusion coefficient (D) calculated by GITT method in charging progress.





**Figure S28.** Rate performance of DPPT ZOBs

**2.3. Theoretical calculations**

**

**

**Figure S29.** UV spectrum of DPPT.





**Figure S30.** UV spectrum of PTO.





**Figure S31.** UV spectrum of DAP.

**
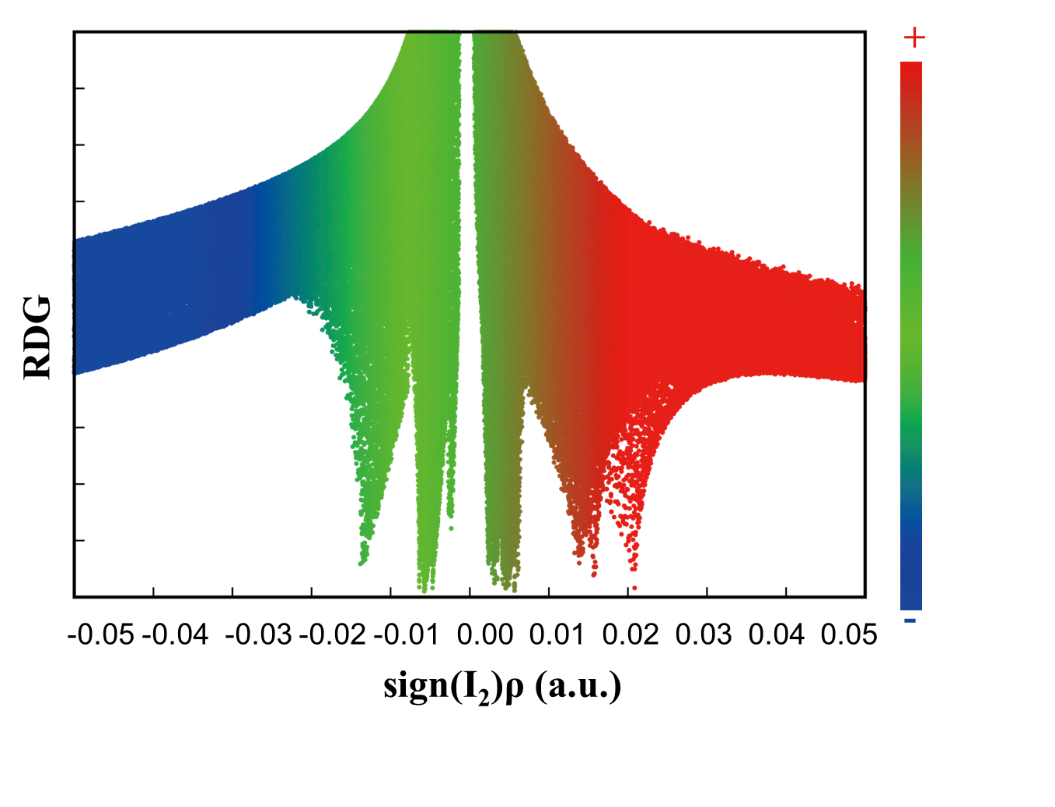
**

**Figure S32.** RDG (Reduced density gradient) scatter maps *vs*. sign(λ_2_)ρ of PTO.

**
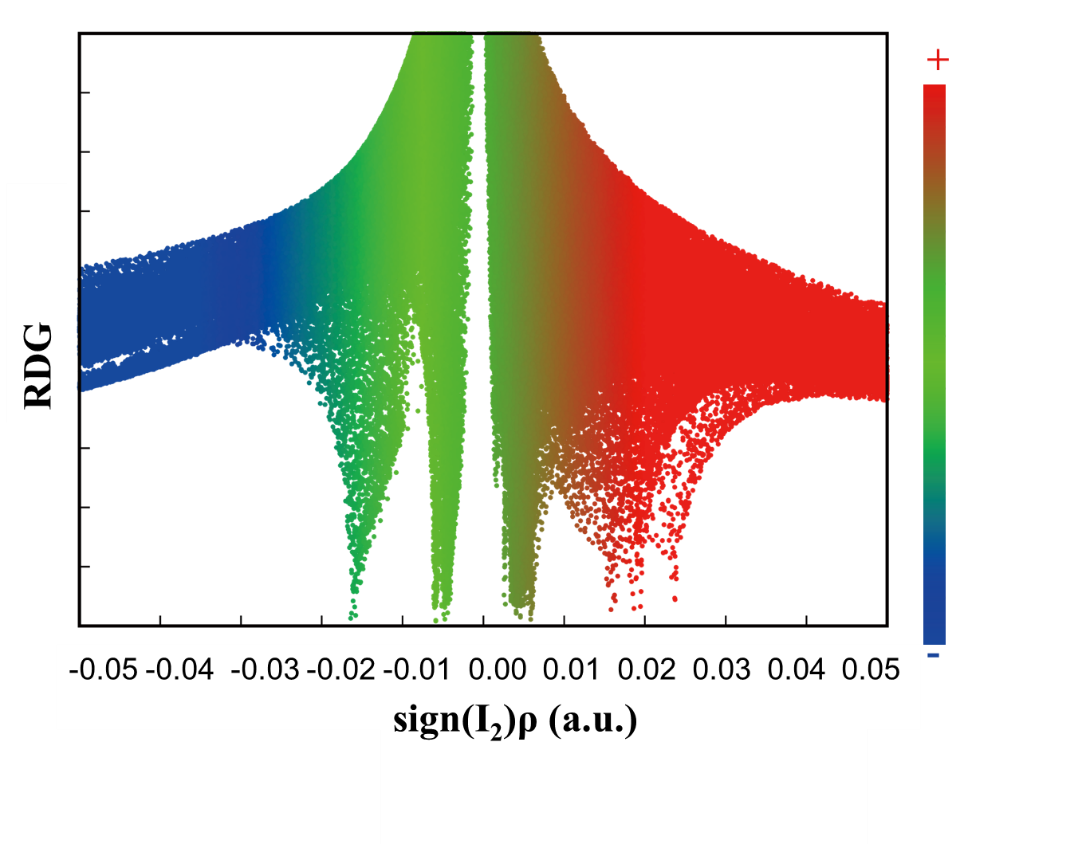
**

**Figure S33.** RDG (Reduced density gradient) scatter maps *vs*. sign(λ_2_)ρ of DAP.


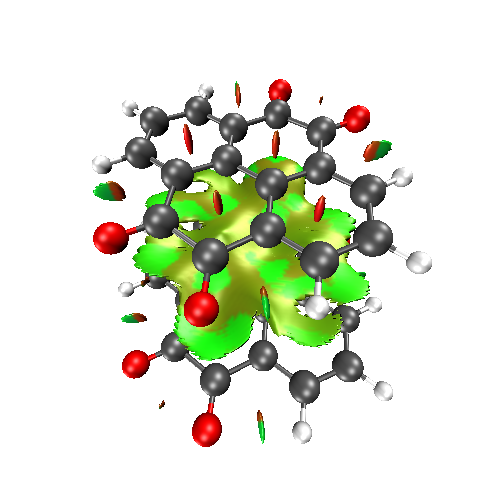


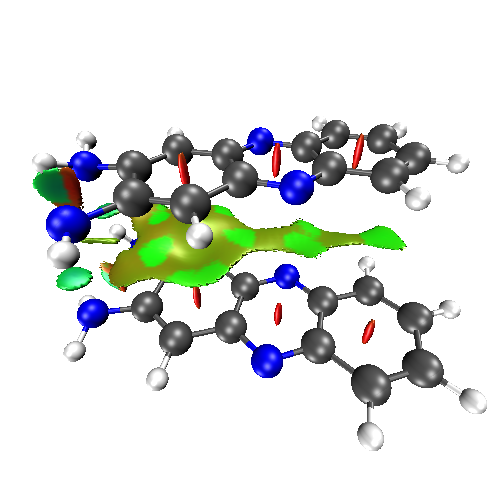


**Figure S34.** RDG visible map of PTO and DAP.


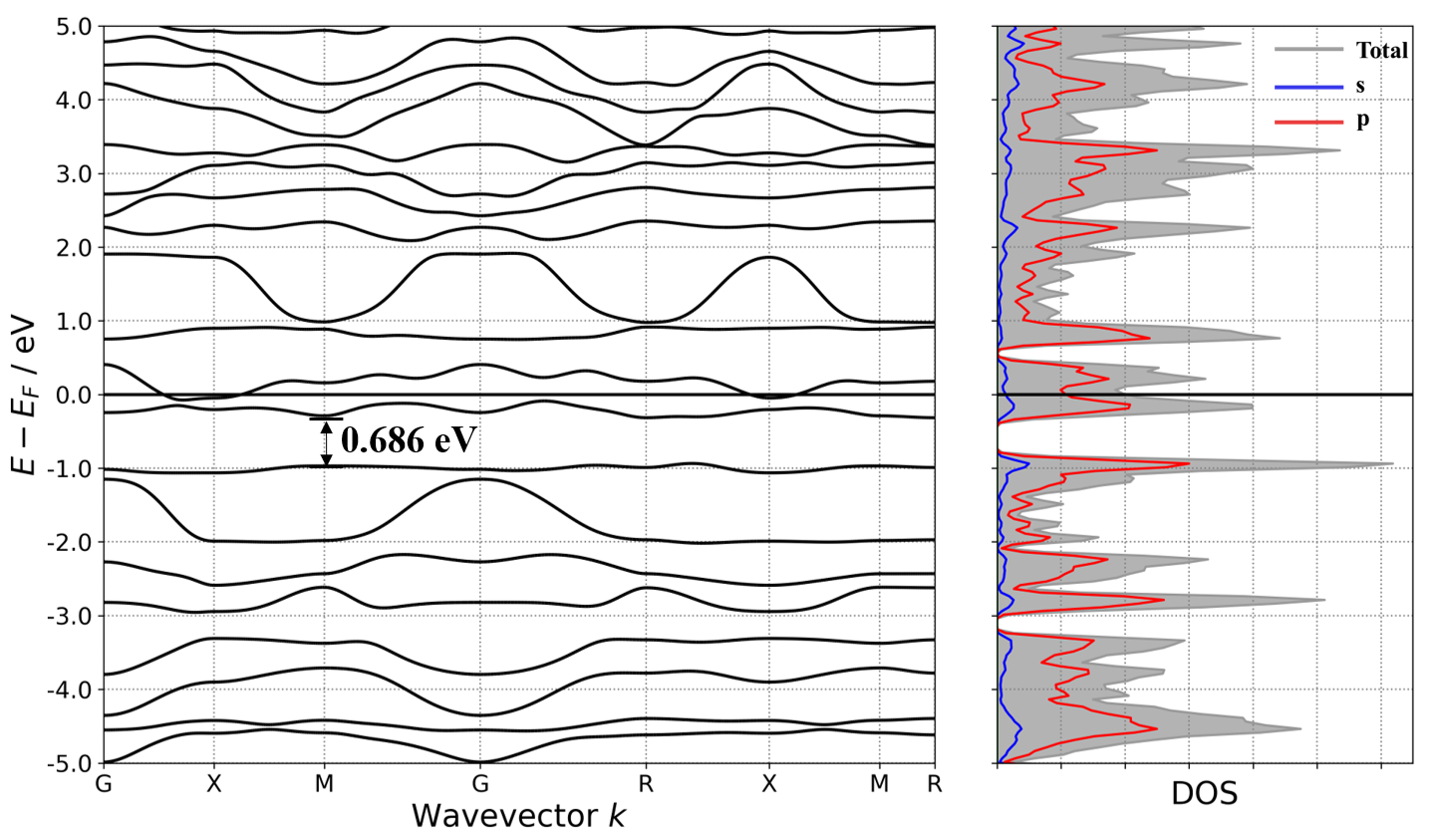


**Figure S35.** Band structure spectrum and corresponding partial density of state (PDOS) of DAP.


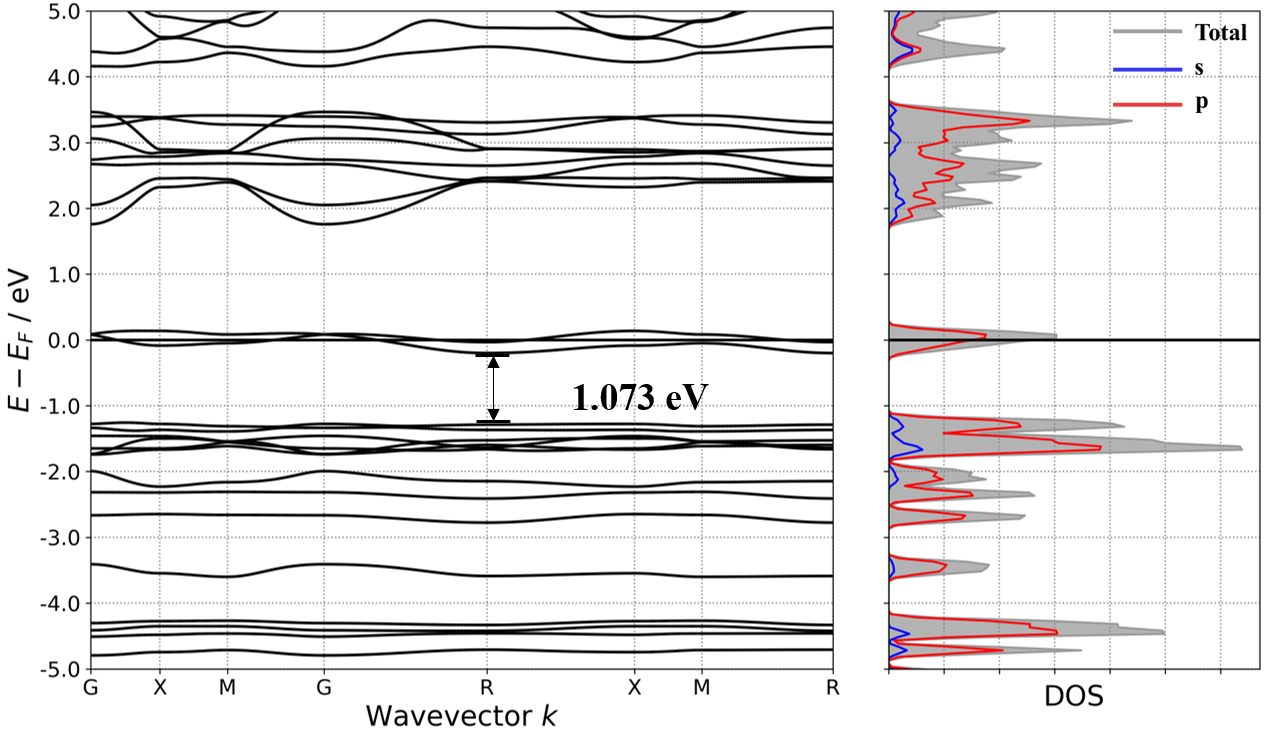


**Figure S36.** Band structure spectrum and corresponding partial density of state (PDOS) of PTO.


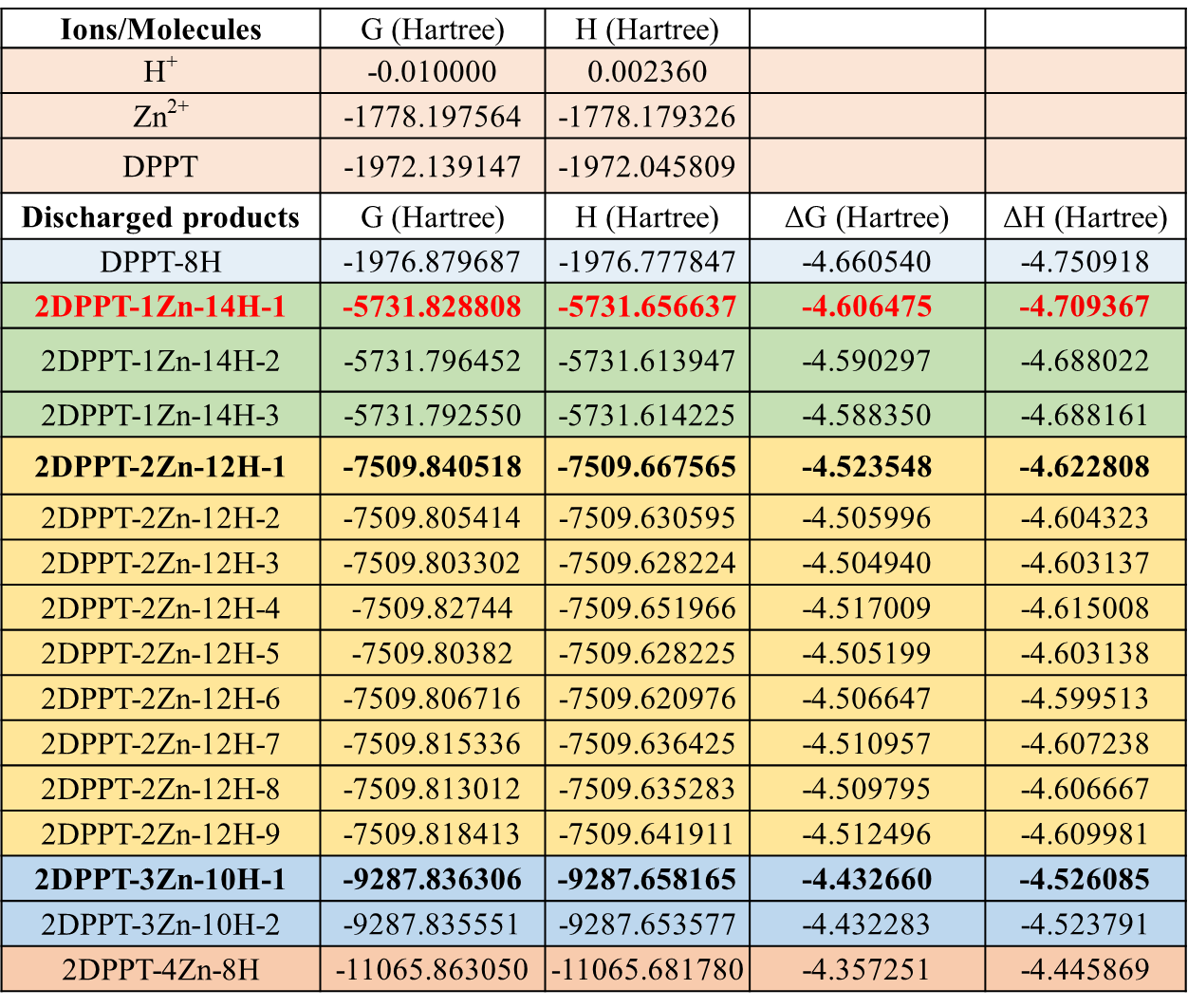


**Figure S37.** Results of calculated G&H, ΔG, ΔH of ions and all possible structures after discharging progress.


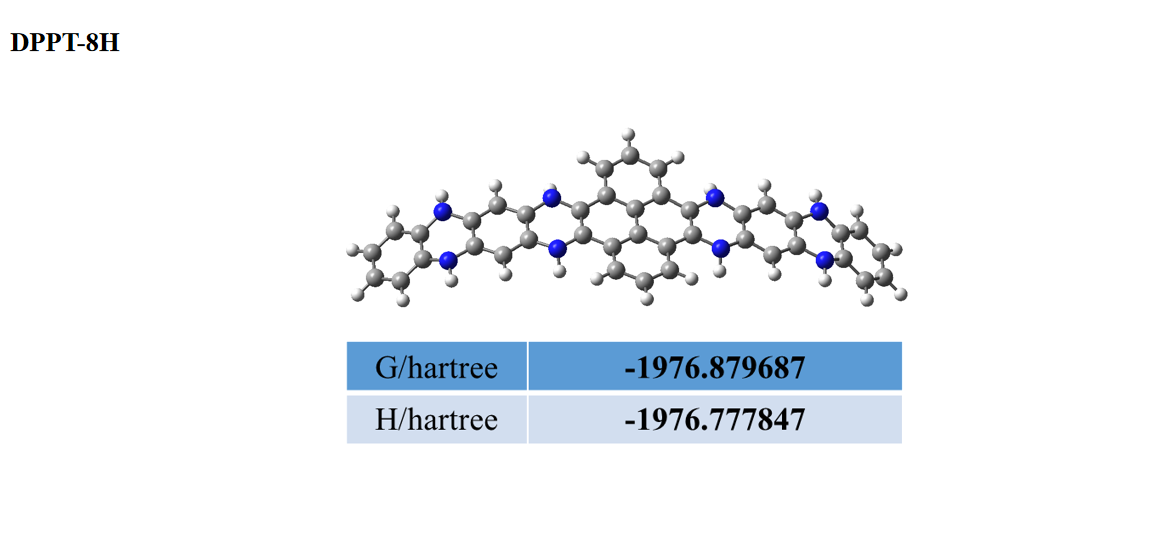


**Figure S38.** Calculated G&H and optimal structure of DPPT-8H.


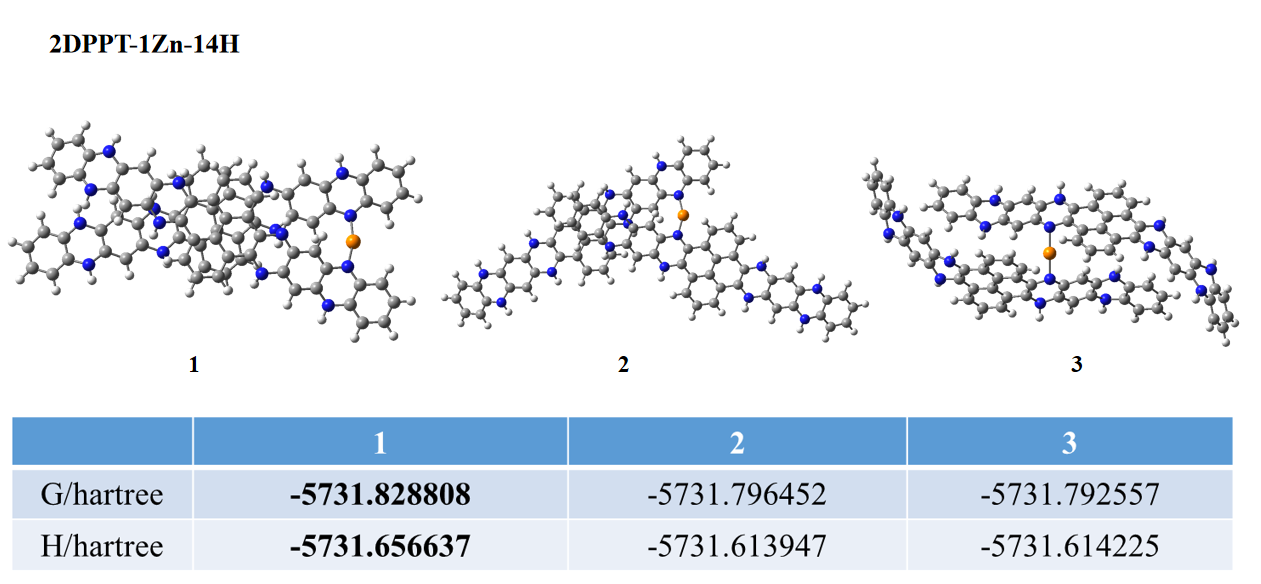


**Figure S39.** Calculated G&H and optimal structure of 2DPPT-1Zn-14H.

**
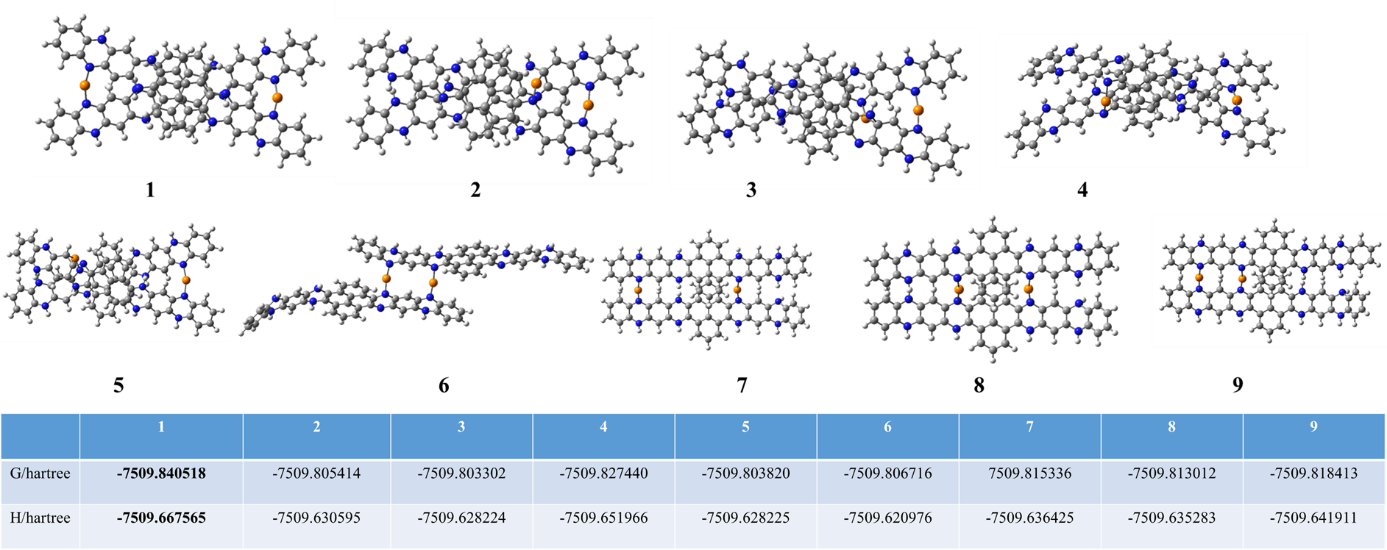
**

**Figure S40.** Calculated G&H and optimal structure of 2DPPT-2Zn-12H.


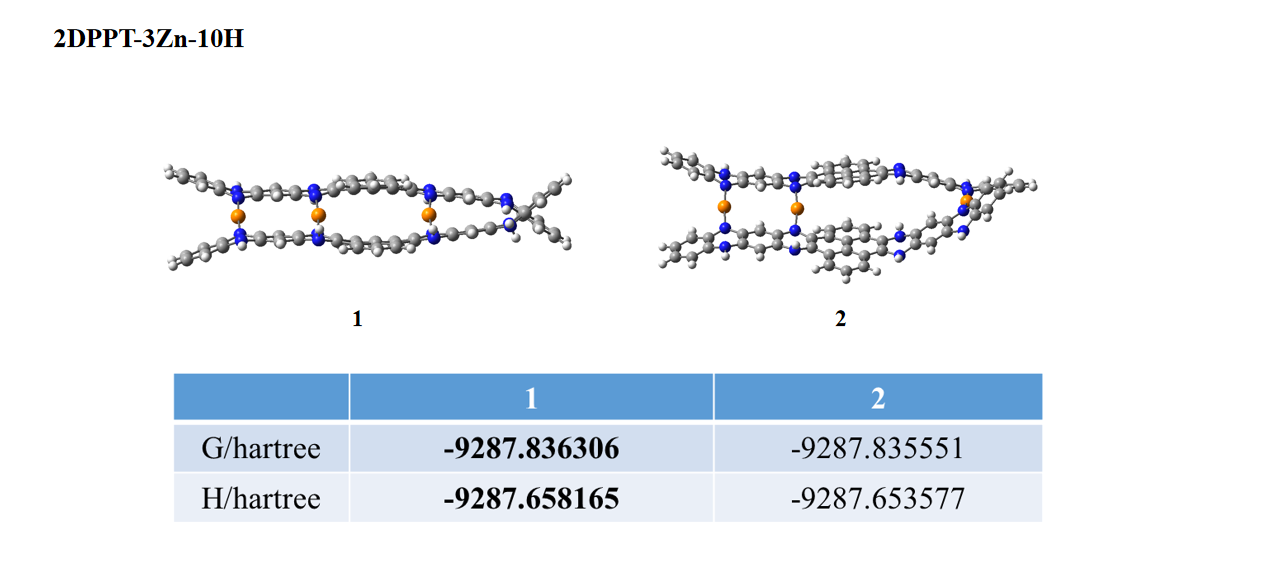


**Figure S41.** Calculated G&H and optimal structure of 2DPPT-3Zn-10H.


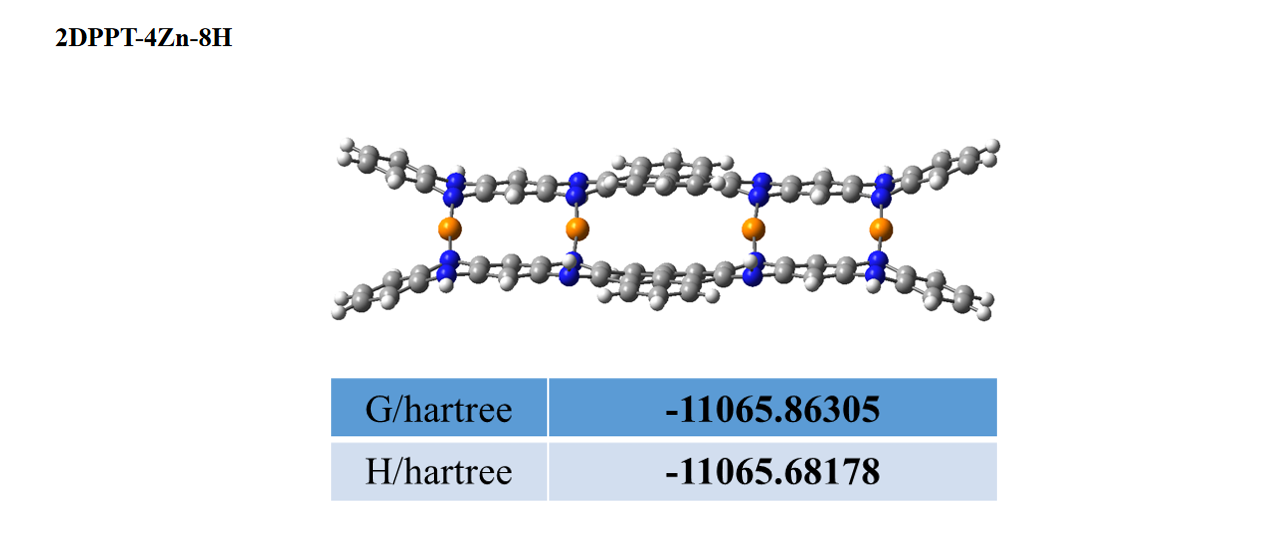


**Figure S42.** Calculated G&H and optimal structure of 2DPPT-4Zn-8H.


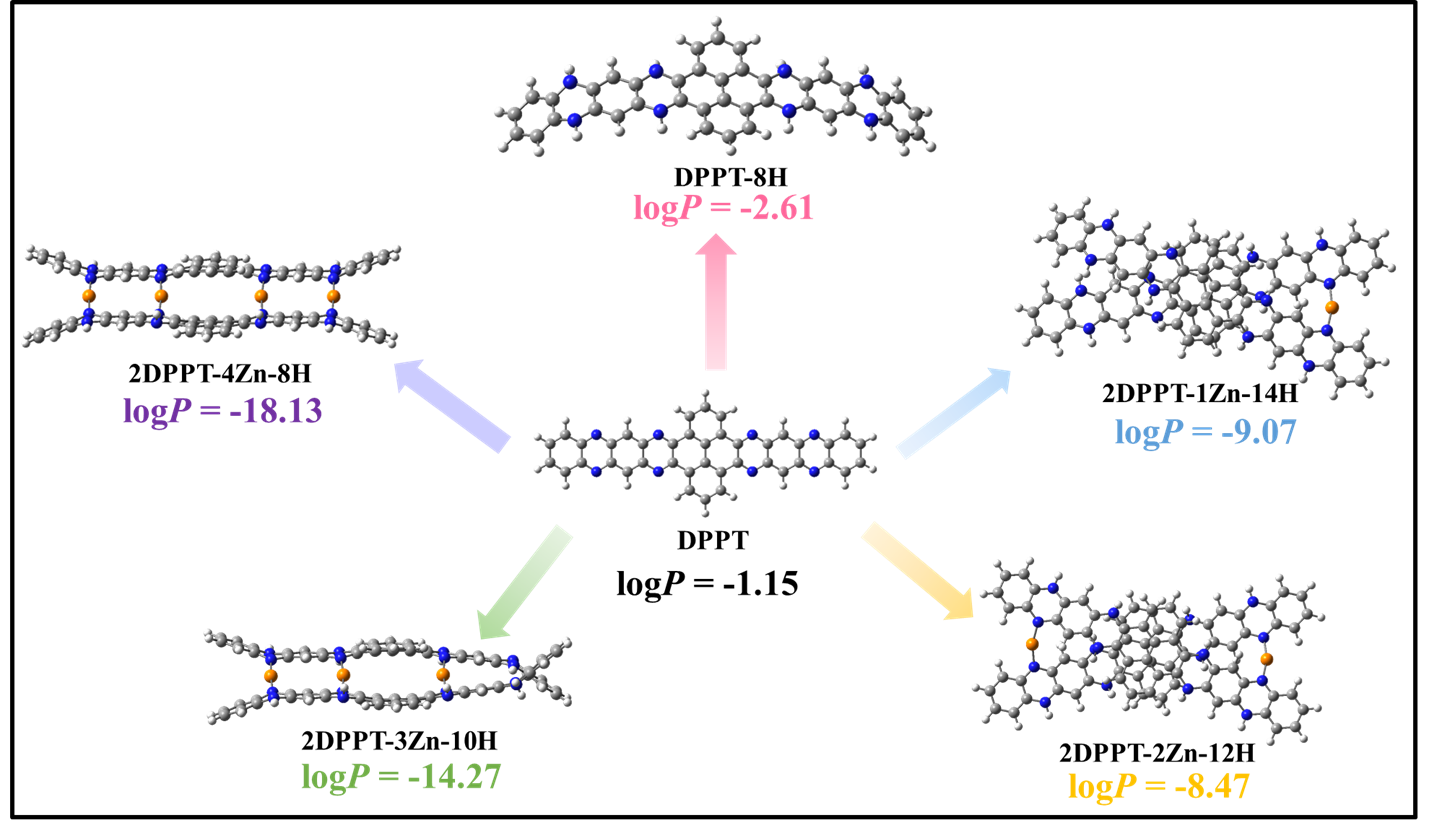


**Figure S43.** The calculated Log*P* of DPPT and discharged products.

2.4. The storage mechanism of DPPT ZOBs during charge/discharge process.





**Figure S44.** CV curves in different electrolytes.

**
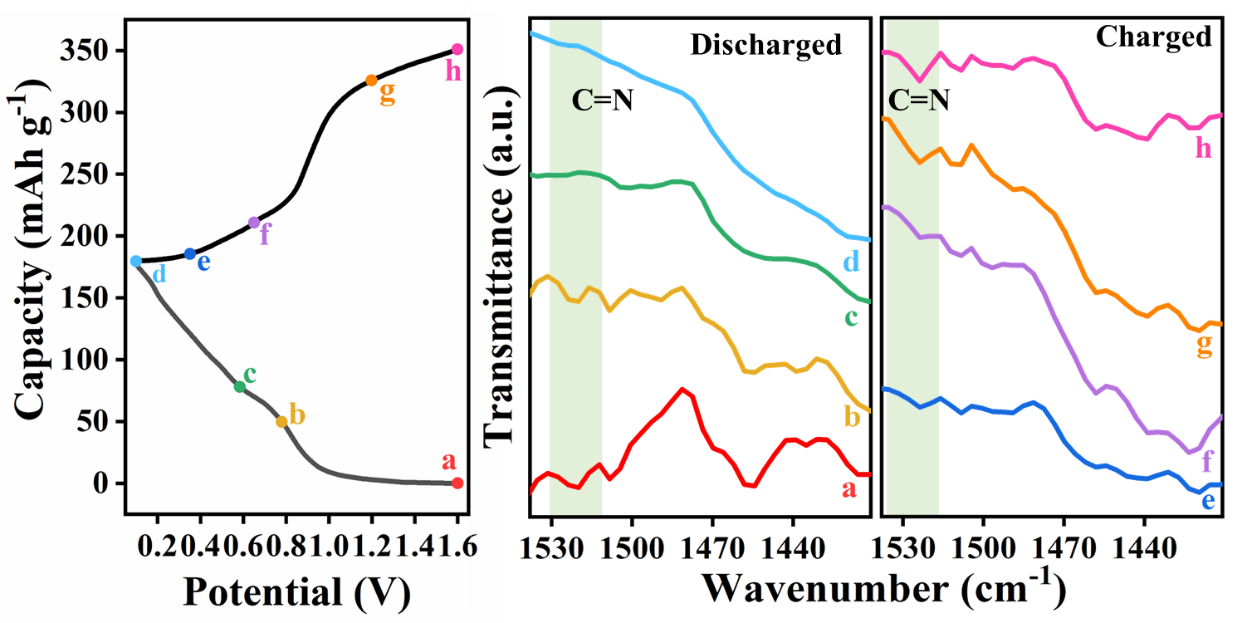
**

**Figure S45.** *Ex-situ* FT-IR spectra of DPPT cathodes at different states.


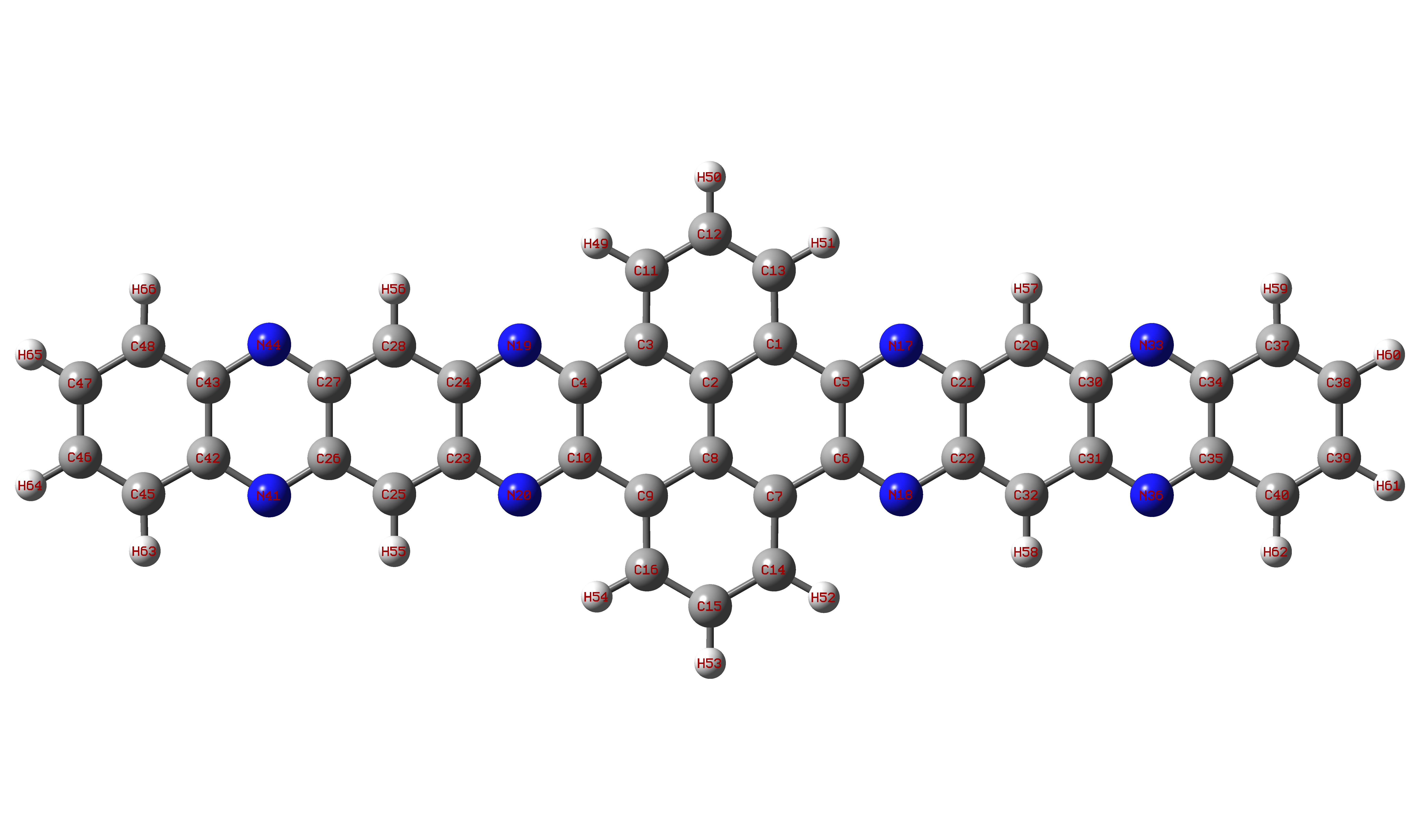

**Figure S46.** Calculated various quantitative indices of DPPT.


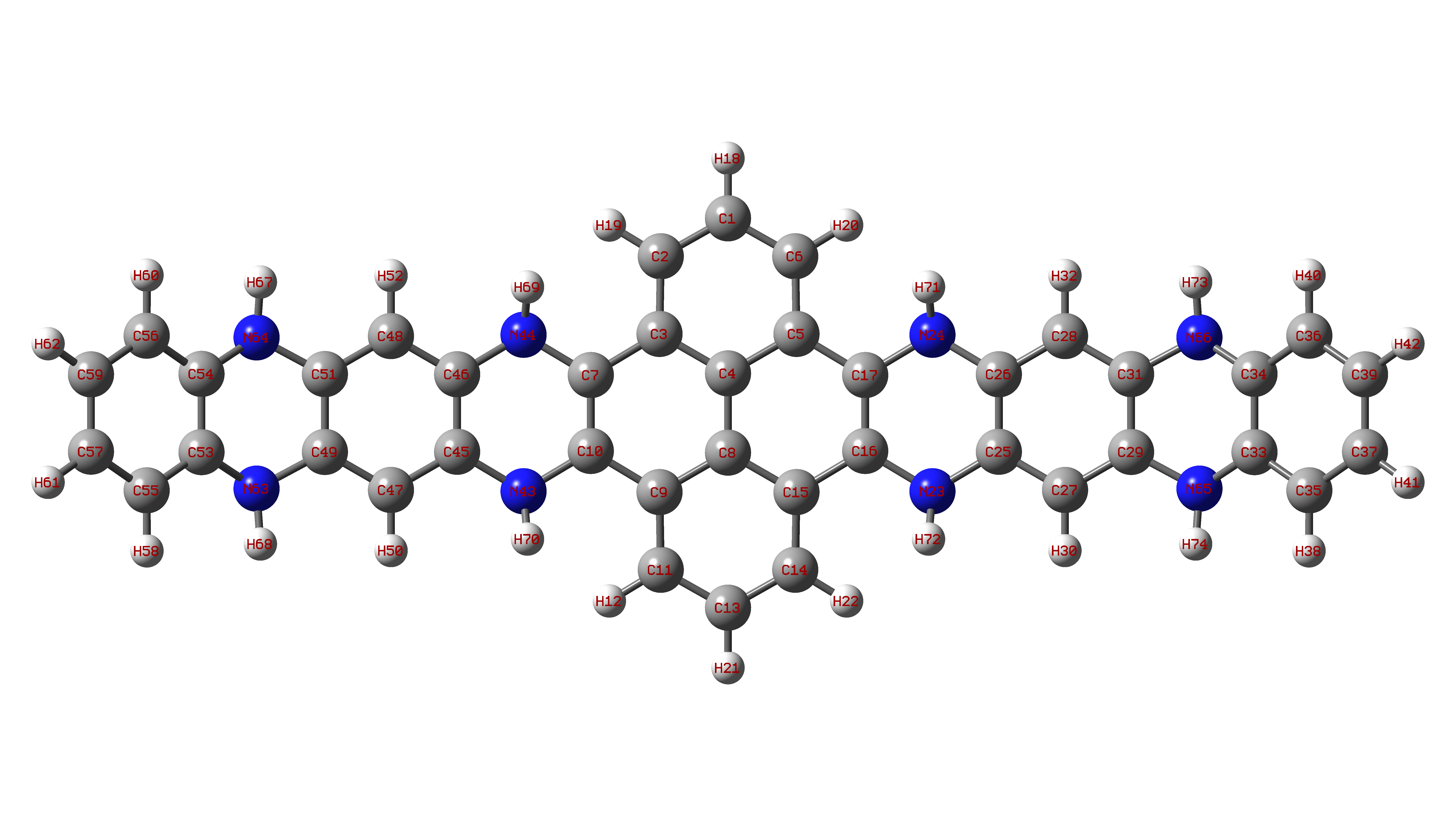

**Figure S47.** Calculated various quantitative indices of DPPT-8H.


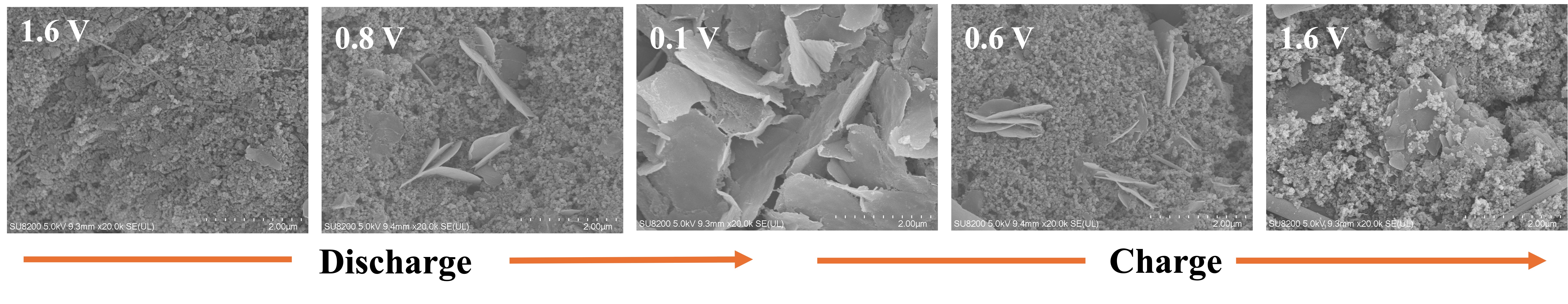


**Figure S48**. The SEM images in the different states at the 1^st^ cycles of DPPT ZOBs.


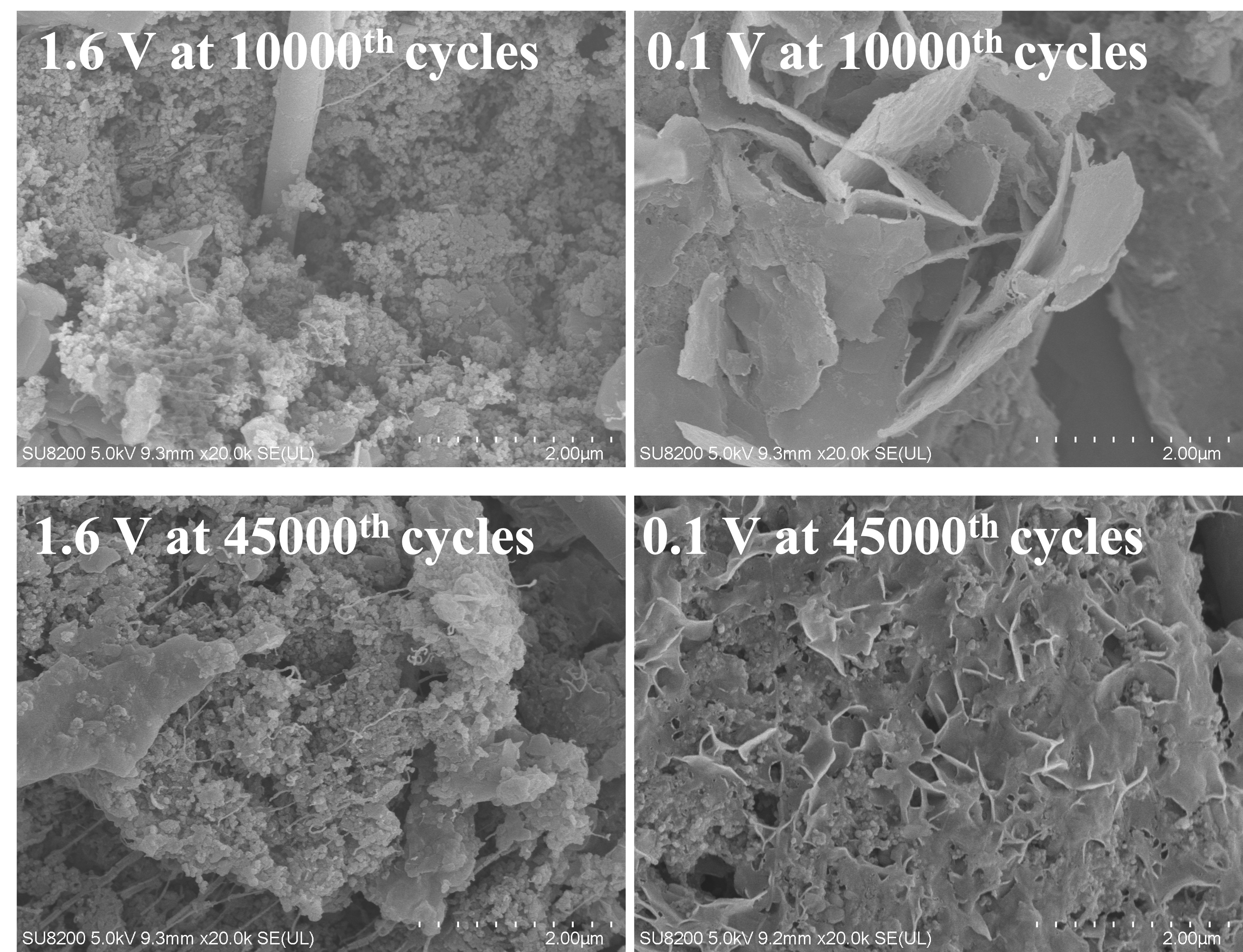


**Figure S49**. The SEM images in the different states at the 10000^th^ and 45000^th^ cycles of DPPT ZOBs.

2.5. Characterizations of flexible DPPT ZOBs and application in wearable NH_3_ sensors.


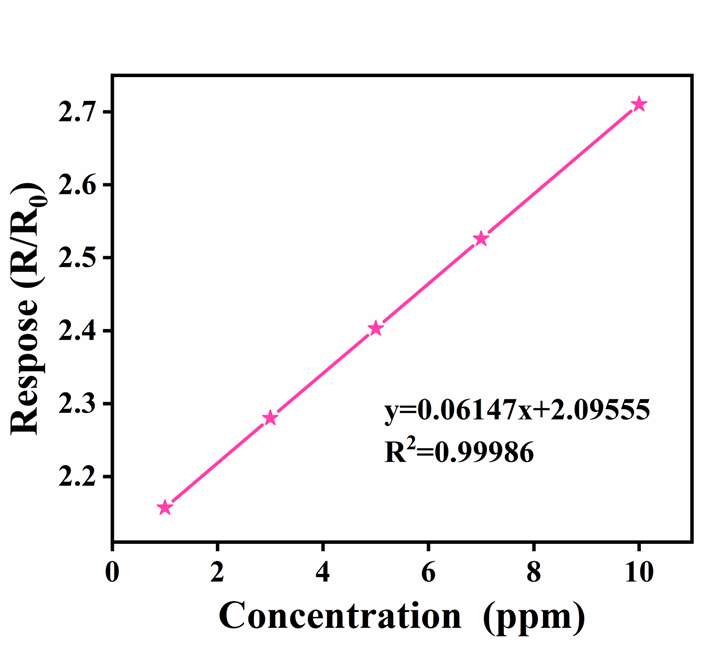


**Figure S50.** The fitting curve of the NH_3_ sensor response value***.***


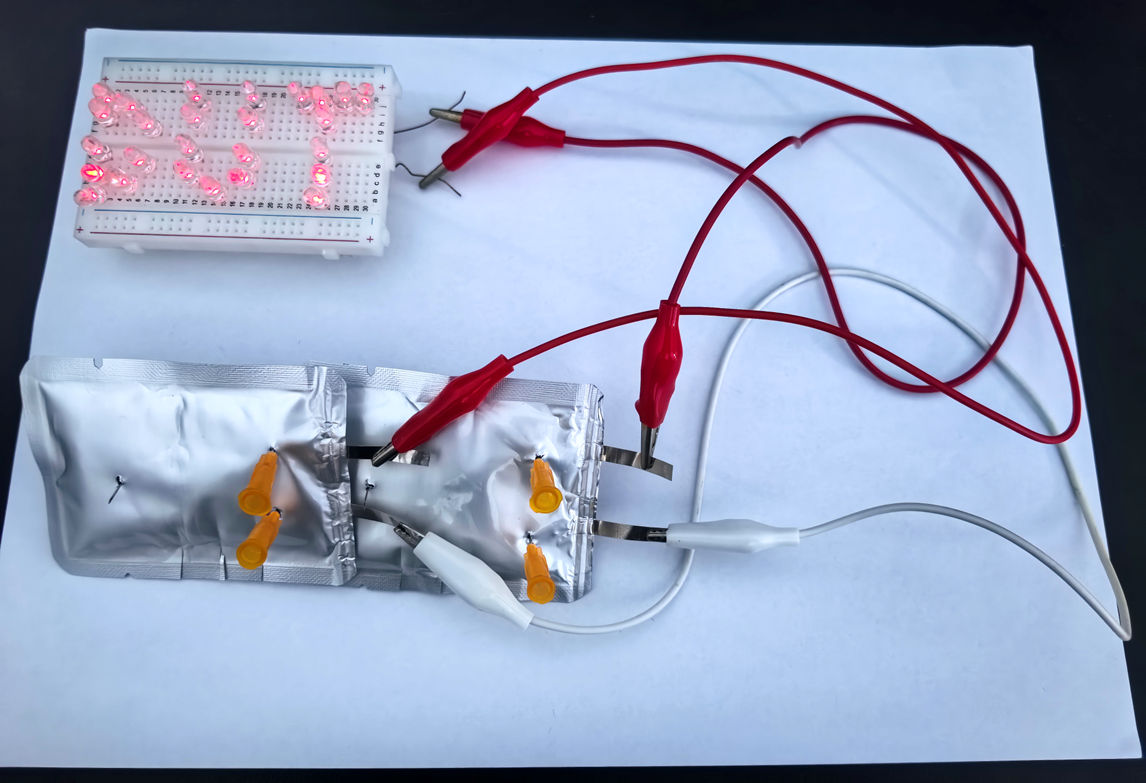


**Figure S51.** Digital photo of the lightened LED lamp powered by flexible DPPT ZOBs after being bent, beaten, drilled, and cut.

**Video S1.** Full-space dynamic streamline field video of DPPT.

**References**

[1] M. J. Frisch, G. W. Trucks, H. B. Schlegel, G. E. Scuseria, M. A. Robb, J. R. Cheeseman, G. Scalmani, V. Barone, G. A. Petersson, H. Nakatsuji, X. Li, M. Caricato, A. V. Marenich, J. Bloino, B. G. Janesko, R. Gomperts, B. Mennucci, H. P. Hratchian, J. V. Ortiz, A. F. Izmaylov, J. L. Sonnenberg, Williams, F. Ding, F. Lipparini, F. Egidi, J. Goings, B. Peng, A. Petrone, T. Henderson, D. Ranasinghe, V. G. Zakrzewski, J. Gao, N. Rega, G. Zheng, W. Liang, M. Hada, M. Ehara, K. Toyota, R. Fukuda, J. Hasegawa, M. Ishida, T. Nakajima, Y. Honda, O. Kitao, H. Nakai, T. Vreven, K. Throssell, J. A. Montgomery Jr., J. E. Peralta, F. Ogliaro, M. J. Bearpark, J. J. Heyd, E. N. Brothers, K. N. Kudin, V. N. Staroverov, T. A. Keith, R. Kobayashi, J. Normand, K. Raghavachari, A. P. Rendell, J. C. Burant, S. S. Iyengar, J. Tomasi, M. Cossi, J. M. Millam, M. Klene, C. Adamo, R. Cammi, J. W. Ochterski, R. L. Martin, K. Morokuma, O. Farkas, J. B. Foresman, D. J. Fox, Wallingford, Gaussian 16 Rev. C.01, Wallingford, CT **2016**.

[2] J.-D. Chai, M. Head-Gordon, Long-range corrected hybrid density functionals with damped atom–atom dispersion corrections. *Physical Chemistry Chemical Physics* **2008**, 10.

[3] T. Lu, F. Chen, A multifunctional wavefunction analyzer. *Journal of Computational Chemistry* **2011**, 33, 580.
